# Supplementary material for: Previous immunity shapes immune responses to SARS-CoV-2 booster vaccination and Omicron breakthrough infection risk
Source: Nat Commun. 2023 Sep 12;14:5624. doi: 10.1038/s41467-023-41342-2 (PMC10497567; doi:10.1038/s41467-023-41342-2)
Supplement: Supplementary file 1 — Supplementary Information [file 41467_2023_41342_MOESM1_ESM.pdf]

Previous immunity shapes immune responses to SARS-CoV-2 booster vaccination and Omicron breakthrough infection risk

Supplementary Information

Table of contents

Supplementary Methods 4

1. Extended experimental methodology 4

1.1. Estimation of anti-receptor-binding domain (RBD) antibody levels 4

1.2. ACE-2/RBD antibody inhibition quantification 4

1.3. T-cell stimulation and IFN-γ quantification 5

2. Data exclusion and data handling before statistical analysis 5

3. Antibody response modeling 6

3.1. Two-part independent modeling of antibody waning and peak projection after boost 6

3.1.1. IgG and neutralizing antibody (nAb) levels – Independent model 6

3.1.1.1. First part of the independent model 6

3.1.1.2. Second part of the independent model 7

3.1.2. IgA responses – Independent model 7

3.1.2.1. First part of the independent model 7

3.1.2.2. Second part of the independent model 7

3.2. IgG antibody levels 8

3.2.1. IgG antibody levels associated with infection status 8

3.2.2. IgG antibody levels associated with reinfection 9

3.2.3. IgG antibody levels associated with future infection 9

3.2.4. IgG antibody levels associated with immune imprinting 9

3.3. Neutralizing antibodies levels 9

3.3.1. Neutralizing antibodies levels associated with infection status 10

3.3.2. Neutralizing antibodies levels associated with reinfection 10

3.3.3. Neutralizing antibodies levels associated with future infection 10

3.3.4. Neutralizing antibodies levels associated with immune imprinting 10

3.4. IgA responses 11

3.4.1. IgA responses associated with infection status 11

3.4.2. IgA responses associated with reinfection 11

3.4.3. IgA responses associated with future infection 11

3.4.4. IgA responses associated with immune imprinting 11

3.5. IFN-γ levels 12

3.5.1. IFN-γ levels associated with infection status 12

3.5.2. IFN-γ levels associated with reinfection 12

3.5.3. IFN-γ levels associated with future infection 12

3.5.4. IFN-γ levels associated with immune imprinting 13

Supplementary Figures 14

Supplementary Figure 1. Dynamics of circulating IgG levels against RBD after the first dose of the BNT162b2 vaccine using two-part independent models. 14

Supplementary Figure 2. Dynamics of circulating neutralizing antibody levels against RBD after the first dose of the BNT162b2 vaccine using two-part independent models. 15

Supplementary Figure 3. Observed and predicted probability of positive IgA responses against RBD after the first dose of the BNT162b2 vaccine using two-part independent models. 16

Supplementary Figure 4. Dynamics of IFN-γ levels released after the first dose of the BNT162b2 vaccine using a linear model. 17

Supplementary Figure 5. Dynamics of circulating IgG levels against RBD after the first dose of the BNT162b2 vaccine in naturally infected individuals before Omicron in relation to reinfection using a non-linear model. 18

Supplementary Figure 6. Dynamics of circulating neutralizing antibody levels against RBD after the first dose of the BNT162b2 vaccine in naturally infected individuals before Omicron in relation to reinfection using a non-linear model. 19

Supplementary Figure 7. Observed and predicted probability of positive IgA responses against RBD after the first dose of the BNT162b2 vaccine naturally infected individuals before Omicron in relation to reinfection using a non-linear model. 20

Supplementary Figure 8. Dynamics of circulating IFN-γ levels released after the first dose of the BNT162b2 vaccine in naturally infected individuals before Omicron in relation to reinfection using a linear model. 21

Supplementary Figure 9. Dynamics of circulating IgG levels against RBD after the first dose of the BNT162b2 vaccine in infection-naïve individuals in relation to future infection using a non-linear model. 22

Supplementary Figure 10. Dynamics of circulating neutralizing antibody levels against RBD after the first dose of the BNT162b2 vaccine in infection-naïve individuals in relation to future infection using a non-linear model. 23

Supplementary Figure 11. Observed and predicted probability of positive IgA responses against RBD after the first dose of the BNT162b2 vaccine in infection-naïve individuals in relation to future infection using a non-linear model. 24

Supplementary Figure 12. Dynamics of IFN-γ levels released after the first dose of the BNT162b2 vaccine in infection-naïve individuals in relation to future infection using a linear model. 25

Supplementary Figure 13. Dynamics of circulating IgG levels against RBD after the first dose of the BNT162b2 vaccine in infected individuals with different immune imprinting status using a non-linear model. 26

Supplementary Figure 14. Dynamics of circulating neutralizing antibody levels against RBD after the first dose of the BNT162b2 vaccine in individuals with different immune imprinting status using a non-linear model. 27

Supplementary Figure 15. Observed and predicted probability of positive IgA responses against RBD after the first dose of the BNT162b2 vaccine in individuals with different immune imprinting status using a non-linear model. 28

Supplementary Figure 16. Dynamics of IFN-γ levels released after the first dose of the BNT162b2 vaccine in individuals infected with different immune imprinting status using a linear model. 29

Supplementary Tables 30
Supplementary Table 1. Demographic data and characteristics of the subgroup study cohorts 30
Supplementary Table 2. Predicted mean IgG levels, represented in AU/ml, in individuals after the administration first vaccination dose according to infection status, age group, and sex 32
Supplementary Table 3. Predicted mean neutralizing antibody levels, represented in IU/ml, in individuals after the administration first vaccination dose according to infection status, age group, and sex 34
Supplementary Table 4. Predicted mean probability of positive IgA responses, represented in percentage, in individuals after the administration first vaccination dose according to infection status, age group, and sex 36
Supplementary Table 5. Predicted mean IFN-γ levels, represented in mIU/ml, in individuals after the administration first vaccination dose according to infection status, age group, and sex 38
Supplementary Table 6. Predicted mean IgG levels, represented in AU/ml, in individuals after the administration first vaccination dose according to reinfection status, age group, and sex 40
Supplementary Table 7. Predicted mean neutralizing antibody levels, represented in IU/ml, in individuals after the administration first vaccination dose according to reinfection status, age group, and sex 42
Supplementary Table 8. Predicted mean probability of positive IgA responses, represented in percentage, in individuals after the administration first vaccination dose according to reinfection status, age group, and sex 44
Supplementary Table 9. Predicted mean IFN-γ levels, represented in mIU/ml, in individuals after the administration first vaccination dose according to reinfection status, age group, and sex 46
Supplementary Table 10. Predicted mean IgG levels, represented in AU/ml, in individuals after the administration first vaccination dose according to future infection status, age group, and sex 47
Supplementary Table 11. Predicted mean neutralizing antibody levels, represented in IU/ml, in individuals after the administration first vaccination dose according to future infection status, age group, and sex 49
Supplementary Table 12. Predicted mean probability of positive IgA responses, represented in percentage, in individuals after the administration first vaccination dose according to future infection status, age group, and sex 51
Supplementary Table 13. Predicted mean IFN-γ levels, represented in mIU/ml, in individuals after the administration first vaccination dose according to future infection status, age group, and sex 53
Supplementary Table 14. Predicted mean IgG levels, represented in AU/ml, in individuals after the administration first vaccination dose according to immune imprinting status, age group, and sex 54
Supplementary Table 15. Predicted mean neutralizing antibody levels, represented in IU/ml, in individuals after the administration first vaccination dose according to immune imprinting status, age group, and sex 56
Supplementary Table 16. Predicted mean probability of positive IgA responses, represented in percentage, in individuals after the administration first vaccination dose according to immune imprinting status, age group, and sex 58
Supplementary Table 17. Predicted mean IFN-γ levels, represented in mIU/ml, in individuals after the administration first vaccination dose according to immune imprinting status, age, and sex 60
Supplementary Table 18. Multiple linear regression analysis for IgG, IgA, neutralizing antibody (nAb), and IFN-γ levels associations with sex, age, time from last vaccine dose administrated or infection, and the infection status in the different cohorts studied (reinfected, future infection, and immune imprinting cohorts). Data are express in log10(AU/ml) for IgG and IgA, log10(IU/ml) for nAb, and log10(mIU/ml) for IFN-γ. 61

Supplementary References 63

Supplementary Methods

1. Extended experimental methodology

1.1. Estimation of anti-receptor-binding domain (RBD) antibody levels

Quantitative estimation of circulating levels of IgG and IgA against ancestral S protein receptor-binding domain (RBD) was evaluated using a national-validated in-house developed ELISA-based assay previously described[1]. Shortly, in-house produced recombinant RBD was coated onto Nunc-Maxisorp 384-well plates (Thermo Fisher Scientific) overnight at 4 °C (1 µg/ml in PBS). The plates were blocked using PBS + 0.05% Tween (Merck) (PBS-T) for 1 hour. Serum samples were diluted accordingly and applied in PBS-T + 5mM EDTA and incubated for 1 hour. RBD-bound antibodies were detected using 0.5 µg/ml HRP-conjugated polyclonal rabbit anti-IgG or IgA (both from Agilent Technologies) and incubated for 1 hour. TMB-One (Kem-En-Tec) was used as a substrate to reveal the plates for 7 min and 10 min for IgG and IgA, respectively. The reaction was stopped by adding 0.3 M H_2SO_4. The calibrator used was a combination of equal concentrations of recombinant human IgG and IgA antibodies against S protein/RBD (1:2000 dilution, both from Genscript). Finally, optical density was measured at 450-630 nm using a Synergy HT absorbance reader (Biotek Instruments). Otherwise stated, all incubations were performed at room temperature shaking. PBS-T was used as washing buffer four times between incubation steps. The threshold for assay positivity was set to 200 and 100 AU/ml for IgG and IgA, respectively, with a sensitivity and specificity of 94.3% and 99.5% and 63.4% and 99.3% for IgG and IgA, respectively[1].

1.2. ACE-2/RBD antibody inhibition quantification

Using an in-house developed pseudo-neutralization ELISA-based assay we estimated the inhibitory capacity of circulating antibodies to prevent the interaction between the host receptor ACE-2 and RBD, as previously described[2]. Briefly, recombinant ACE-2 produced in-house was coated onto Nunc Maxisorp 96-well plates (Thermo Fisher Scientific) overnight at 4 °C (1 µg/ml in PBS). In Nunc low-binding round-bottom 96-well plates (Thermo Fisher Scientific) serum samples were diluted accordingly and co-incubated with a solution containing HRP-conjugated high-sensitivity streptavidin (dilution 1:16000, Pierce) and biotinylated recombinant RBD (4 ng/ml, in-house produced) in PBS-T for 1 hour. Following this, the mixture was transferred to the ACE-2-coated plates and incubated for 35 minutes. The plates were developed for 20 min as described above. Otherwise stated, all incubations were performed at room temperature shaking. PBS-T was used as washing buffer four times between incubation streps. The threshold for assay positivity was set to 420 IU/ml with a sensitivity and specificity of 99.5% sensitivity and 98.8% specificity. This pseudo-

neutralizing assay has a great correlation (r=0.9231) with the gold standard plaque reduction neutralization test[2].

1.3. T-cell stimulation and IFN-γ quantification

T-cells in whole blood were specifically stimulated with S1-based peptides using the SARS-CoV-2 IGRA stimulation tube set from EUROIMMUN (ET 2606-3003) following manufacturer's instructions. Shortly, heparinized whole blood (0.5 ml) was aliquoted into the stimulation tubes provided by the kit: a) a blank tube to quantify the unspecific stimulation of T-cells and subsequent IFN-γ release, b) a specific tube containing S1-based peptides, and c) a positive tube containing a mitogen. Tubes containing blood were inverted six times and incubated for 21 hours at 37 °C. Thereafter, the reaction was stopped by centrifugation at 12,000 g for 10 min. The plasma mas collected and stored (- 80 °C) until quantification of IFN-γ released.

IFN-γ released from T-cells upon stimulation was quantified using the IFN-γ ELISA kit from EUROIMMUN (ET 6841-9601) following manufacturer's instructions. Shortly, diluted samples were applied into the pre-coated plates in sample buffer and incubated for 2 hours. The standard curve and the controls provided by the kits were applied undiluted. Bound IFN-γ was detected by incubating for 30 minutes a biotin-labeled antibody against human IFN-γ followed by 30 minutes incubation with peroxidase-labelled streptavidin. Plates were developed for 20 min using TMB as a substrate and the reaction was stopped by adding 0.5 M H_2SO_4. Optical density was measured as described above. Washing buffer was used for washing the plates between incubation steps (five times). Incubation steps were performed without shaking at room temperature. The assay positivity threshold set by the manufacturer was 200 mIU/ml.

2. <u>Data exclusion and data handling before statistical analysis</u>

The baseline sample collected from individuals who have had a natural infection more than 14 days before the administration of the first vaccine dose was excluded from the analyses. The days from baseline to the vaccine in infection-naïve individuals with a baseline sample collected before the administration of the first dose was reset to 0. Individuals vaccinated with a different vaccine to BNT162b2 were excluded from the analyses (n=124). Regarding statistical analyses to evaluate associations with SARS-CoV-2 reinfection, individuals who had a first infection with the virus after the administration of two doses of the BNT162b2 vaccine were excluded due to the lack of homogeneity in the antibody dynamics before the waning period after the administration of the second dose. A total of 14 individuals were excluded due to the uncertainty to identify whether the individual was infected with a variant earlier to Omicron or with Omicron. Individuals who did not receive the third dose were excluded from the study (n=4). R packages used in this study were

readxl (version 1.4.1), dplyr (version 1.0.9), tidyr (version 1.2.0), lubridate (version 1.8.0), writexl (version 1.4.0), glmmTMB (version 1.1.4), DHARMa (version 0.4.5), lmerTest (version 3.1.3), car (version 3.1.0), carData (version 3.0.5), lme4 (version 1.1.30), Matrix (version 1.3.3), ggplot2 (version 3.3.6), reshape (version 0.8.9), ggpubr (version 0.4.0), wesanderson (version 0.3.6), ggthemes (version 4.2.4), and ggnewscale (version 0.4.7).

3. Antibody response modeling

Antibody levels and responses over time were evaluated using generalized mixed models with and without cubic splines included in the R package glmmTMB[3]. The R package DHARMa (https://cran.r-project.org/web/packages/DHARMa/index.html) was employed for all the mixed models to check the model fitness and to evaluate diagnostics of the residuals. The models used in this study were described in a previous publication by our group[4] and modified accordingly to fit the new study cohort.

3.1. Two-part independent modeling of antibody waning and peak projection after boost

Due to the study design, there was insufficient observed data between the 6-month and 12-month round to evaluate the antibody decline until boost administration and the peak generated after this event. To evaluate this, we fitted a two-part independent model, the first part consisted of the modelling of the prime dynamics and the second part modelling of the boost dynamics.

3.1.1. IgG and neutralizing antibody (nAb) levels – Independent model

3.1.1.1. First part of the independent model

Log10-transformed IgG/nAbs levels were fitted in the zero-inflated Gaussian mixed model with subject-level random effects for both intercept and slope across days from first vaccination dose. Days from first vaccination dose were represented with two natural cubic splines (NCS), allowing the modeling of non-linear trends. The knots of the two NCS were defined based on median of the days from first vaccination at 21-day and the peak reached after 2-month sample collection times. Alongside the two NCS, infection status, age groups and sex were fitted in the model. Moreover, a two-way interaction between NCS and infection status, NCS and age groups, and between infection status and age groups (only for IgG) were included to evaluate IgG/nAbs dynamics over time. Days from first vaccination dose, infection status, and sex were included as

independent variables for the zero-inflated model. This model was used to project the IgG/nAbs levels until day 295 from the first vaccine dose (median days of boost administration).

3.1.1.2. Second part of the independent model

Log10-transformed IgG/nAbs levels were fitted in the linear mixed model with subject-level random effect for both intercept and slope across days from the first vaccination dose. Variables included in the linear mixed model were days from first vaccination dose, infection status, age groups, and sex. Moreover, a two-way interaction between days from the first vaccine and infection status, days from the first dose and age groups, and between infection status and age groups (only for IgG) were included to evaluate the IgG/nAbs dynamics over time. This model was used to project the IgG/nAbs levels from day 309 from the first vaccine dose.

Predicted data from the first and the second part of the independent models were merged and used to plot the IgG/nAbs dynamics over time.

3.1.2. IgA responses – Independent model

3.1.2.1. First part of the independent model

Binary responses of positive IgA responses were fitted using a GLMM with binomial distribution and with subject-level random effects for intercept. Days from first vaccination dose were represented with two NCS, allowing the modeling of non-linear trends. The knots of the two NCS were defined based on median of the days from first vaccination at 21-day and the peak reached after 2-month sample collection times. Alongside the two NCS, infection status, age groups, and sex were fitted in the model. Moreover, a two-way interaction between NCS and infection status was included to evaluate the IgA responses over time. This model was used to predict the probability of positive IgA responses until day 295 from the first vaccine dose (median days of boost administration).

3.1.2.2. Second part of the independent model

Binary responses of positive IgA responses were fitted in the GLMM with subject-level random effect for both intercept and slope across days from the first vaccination dose. Variables included in the GLMM were days from first vaccination dose, infection status, age groups, and sex. Moreover, a two-way interaction

between days from the first vaccine and infection status was included to evaluate the IgA responses dynamics over time. This model was used to project the probability of positive IgA responses from day 309 from the first vaccine dose.

Predicted data from the first and the second part of the independent models were merged and used to plot the IgA responses dynamics over time.

3.2. IgG levels

Zero-inflated Gaussian mixed models were employed to evaluate the levels of IgG as the baseline levels presented a high proportion of samples with non-detectable IgG. This correction was necessary to assume the normal distribution of the IgG levels data. Therefore, the zero-inflated Gaussian mixed model consisted of two parts, one based on a conditional model that fitted a Gaussian mixed model for the detectable IgG levels (also referred as non-zero observations), and another part that based on a zero-inflated model that fitted a binary model for the detectable vs non-detectable IgG levels (fitting the probability of observing a zero observation or not).

Log10-transformed IgG levels were fitted in the zero-inflated Gaussian mixed model with subject-level random effects for both intercept and slope across days from first vaccination dose. Days from first vaccination dose were represented with five NCS, allowing the modeling of non-linear trends. The knots of the five NCS were defined based on median of the days from first vaccination at 21-day, 2-month, 6-month, and 12-month sample collection times.

3.2.1. IgG levels associated with infection status

Alongside the five NCS, infection status, age groups and sex were fitted in the model. Moreover, a two-way interaction between NCS and infection status, NCS and age groups, NCS and sex, infection status and age groups, and between infection status and sex were included to evaluate IgG dynamics over time. Days from first vaccination dose, infection status, and sex were included as independent variables for the zero-inflated model.

3.2.2. IgG levels associated with reinfections

Alongside the five NCS, reinfection status, age groups and sex were fitted in the model. Moreover, a two-way interaction between NCS and reinfection status was included to evaluate IgG dynamics over time. Interactions between NCS and age groups, and NCS and sex were not included because of the lack of significance.

3.2.3. IgG levels associated with future infections

Alongside the five NCS, future infection status, age groups, and sex were fitted in the model. Moreover, a two-way interaction between NCS and future infection status, NCS and age groups, and NCS and sex were included to evaluate IgG dynamics over time. Days from first vaccination dose was included as independent variable for the zero-inflated model.

3.2.4. IgG antibody levels associated with immune imprinting

Alongside the five NCS, immune imprinting status, age groups, and sex were fitted in the model. Moreover, a two-way interaction between NCS and immune imprinting status, NCS and age groups, and NCS and sex were included to evaluate IgG dynamics over time. Days from first vaccination, immune imprinting status, and sex were included as independent variables for the zero-inflated model.

3.3. Neutralizing antibodies levels

Zero-inflated Gaussian mixed models were employed to evaluate the levels of neutralizing antibodies as the baseline levels presented a high proportion of samples with non-detectable neutralizing antibodies. This correction was necessary to assume the normal distribution of the neutralizing antibodies levels data. The zero-inflated Gaussian mixed model consisted of two parts, one based on a conditional model that fitted a Gaussian mixed model for the detectable neutralizing antibodies levels (also referred as non-zero observations), and another part that was based on a zero-inflated model that fitted a binary model for the detectable vs non-detectable neutralizing antibodies levels (fitting the probability of observing a zero observation or not).

Log10-transformed neutralizing antibodies levels were fitted in the zero-inflated Gaussian mixed model with subject-level random effects for both intercept and slope across days from first vaccination dose. Days from first vaccination dose were represented with five natural cubic splines (NCS), allowing the modeling of non-

linear trends. The knots of the five NCS were defined based on median of the days from first vaccination at 21-day, 2-month, 6-month, and 12-month sample collection times.

3.3.1. Neutralizing antibodies levels associated with infection status

Alongside the five NCS, infection status, age groups and sex were fitted in the model. Moreover, a two-way interaction between NCS and infection status, NCS and age groups, and NCS and sex were included to evaluate neutralizing antibodies dynamics over time. Interaction between infection status and age groups, and infection status and sex were not included because of the lack of significance. Days from first vaccination dose was included as independent variable for the zero-inflated model.

3.3.2. Neutralizing antibodies levels associated with reinfections

For this analysis, the zero-inflated model was not included as the proportion of non-detectable neutralizing antibodies was low in this subgroup.

Alongside the five NCS, reinfection status, age groups, and sex were fitted in the model. Moreover, a two-way interaction between NCS and reinfection status was included to evaluate neutralizing antibodies dynamics over time. Interaction between NCS and age groups or sex were not included because of the lack of significance.

3.3.3. Neutralizing antibodies levels associated with future infections

Alongside the five NCS, future infection status, age groups, and sex were fitted in the model. Moreover, a two-way interaction between NCS and future infection status, NCS and age groups, and NCS and sex were included to evaluate IgG dynamics over time.

3.3.4. Neutralizing antibodies levels associated with immune imprinting

Alongside the five NCS, immune imprinting status, age groups and sex were fitted in the model. Moreover, a two-way interaction between NCS and immune imprinting status was included to evaluate neutralizing antibodies dynamics over time. Interaction between NCS and age groups, and NCS and sex were not included due to the sample size.

3.4. IgA responses: Binomial generalized mixed model

Binomial generalized mixed models were used to model IgA responses over time after first vaccination dose. Binary responses of positive IgA responses with subject-level random effects for intercept were fitted. Days from first vaccination dose were represented with five NCS, allowing the modeling of non-linear trends. The knots of the five NCS were defined based on median of the days from first vaccination at 21-day, 2-month, 6-month, and 12-month sample collection times.

3.4.1. IgA responses associated with infection status

Alongside the five NCS, infection status, age groups, and sex were fitted in the model. Moreover, a two-way interaction between NCS and infection status, and NCS and age groups were included to evaluate the IgA responses over time. Interaction between NCS and sex was not included because of the lack of significance.

3.4.2. IgA responses associated with reinfection

Alongside the five NCS, reinfection status, age groups, and sex were fitted in the model. Moreover, a two-way interaction between NCS and reinfection status, and NCS and age groups were included to evaluate the IgA responses over time. Interaction between NCS and sex was not included because of the lack of significance.

3.4.3. IgA responses associated with future infection

Alongside the five NCS, future infection status, age groups, and sex were fitted in the model. Moreover, a two-way interaction between NCS and future infection status was included to evaluate the IgA responses over time. Interactions between NCS and age groups, and NCS and sex were not included because of the lack of significance.

3.4.4. IgA responses associated with immune imprinting

Alongside the five NCS, future immune imprinting status, age groups, and sex were fitted in the model. Moreover, a two-way interaction between NCS and immune imprinting status was included to evaluate the IgA responses over time. Interactions between NCS and age groups, and NCS and sex were not included because of the lack of significance.

3.5. IFN-γ levels

Linear mixed models were employed to model IFN-γ levels over time from after first vaccination. Log10-transformed IFN-γ levels were fitted in the linear mixed model with subject-level random effects for both intercept and slope across days from first vaccination.

A zero-inflated linear mixed model was employed to evaluate levels of IFN-γ associated with infection status as the baseline levels presented a high proportion of samples with non-detectable IFN-γ levels. This correction was necessary to assume the normal distribution of the IFN-γ levels data. The zero-inflated Gaussian mixed model consisted of two parts, one based on a conditional model that fits a Gaussian mixed model for the detectable IFN-γ levels (also referred as non-zero observations), and another part that was based on a zero-inflated model that fits a binary model for the detectable vs non-detectable IFN-γ levels (fitting the probability of observing a zero or not).

3.5.1. IFN-γ levels associated with infection status

Variables included in the linear mixed model were days from first vaccination dose, infection status, age groups, and sex. Moreover, a two-way interaction between days from the first vaccine and infection status was included to evaluate the IFN-γ dynamics over time. Infection status was included as independent variable for the zero-inflated model.

3.5.2. IFN-γ levels associated with reinfection

The zero-inflated part was not included in this analysis as the proportion of non-detectable IFN-γ levels (also referred as zero observations) was low.

Variables included in the linear mixed model were days from first vaccination dose, reinfection status, age groups, and sex. Moreover, a two-way interaction between days from the first vaccine and reinfection status was included to evaluate the IFN-γ dynamics over time.

3.5.3. IFN-γ levels associated with future infection

Variables included in the linear mixed model were days from first vaccination dose, future infection status, age groups, and sex. Moreover, a two-way interaction between days from the first vaccine and future infection status was included to evaluate the IFN-γ dynamics over time.

3.5.4. IFN-γ levels associated with immune imprinting

Variables included in the linear mixed model were days from first vaccination, immune imprinting status, age groups, and sex. Moreover, a two-way interaction between days from the first vaccine and immune imprinting status was included to evaluate the IFN-γ dynamics over time.

Supplementary Figures

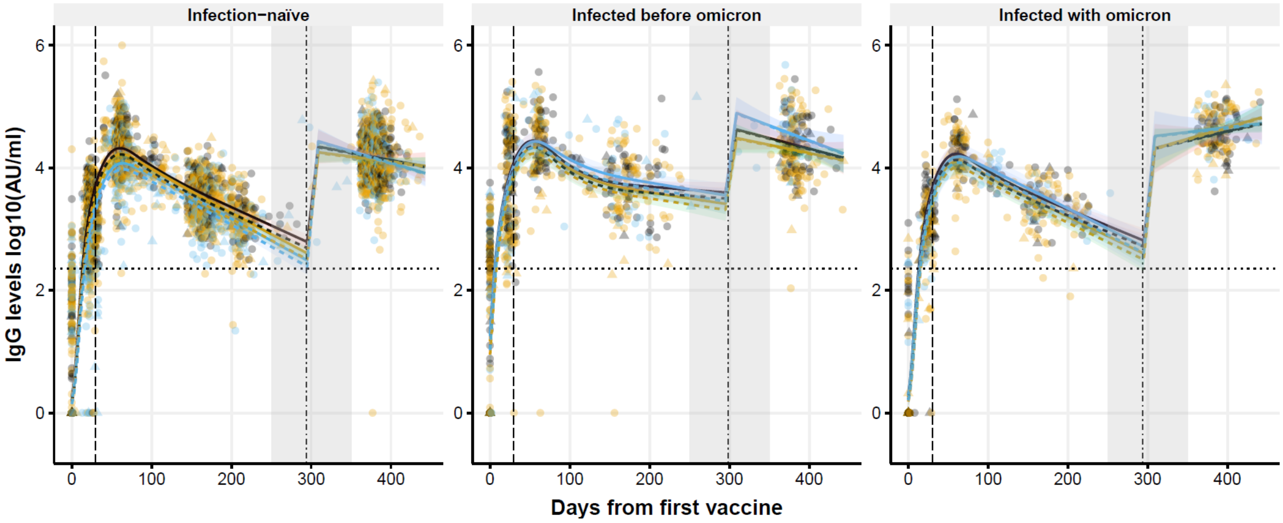

Supplementary Figure 1. Dynamics of circulating IgG levels against RBD after the first dose of the BNT162b2 vaccine using two-part independent models. Distribution of IgG levels, represented in log10(AU/ml), over time (days from the first vaccine) in infection-naïve individuals (left), in individuals previously infected with a variant before Omicron (middle), and in individuals infected with Omicron (right). Circles and triangles represent the observed levels of circulating IgG levels in females and males, respectively. Solid and dashed lines represent the predicted levels of circulating IgG levels calculated by the model in females and males, respectively. Black, yellow, and blue colors represent individuals with age <40, 40–60, and >60 years, respectively. Horizontal black dotted line represents the threshold for assay positivity. Vertical dashed and dashed-dotted lines represent when the second and the third dose was administered, respectively (median days). Shadowed areas represent the 95% confidence interval. Centre for the confidence interval is the predicted (mean) values. Predictive values in the grey-shaded area (days 250–350) do not represent a realistic projection due to insufficient observed data to provide realistic predictive data. Source data are provided as a Source Data file.

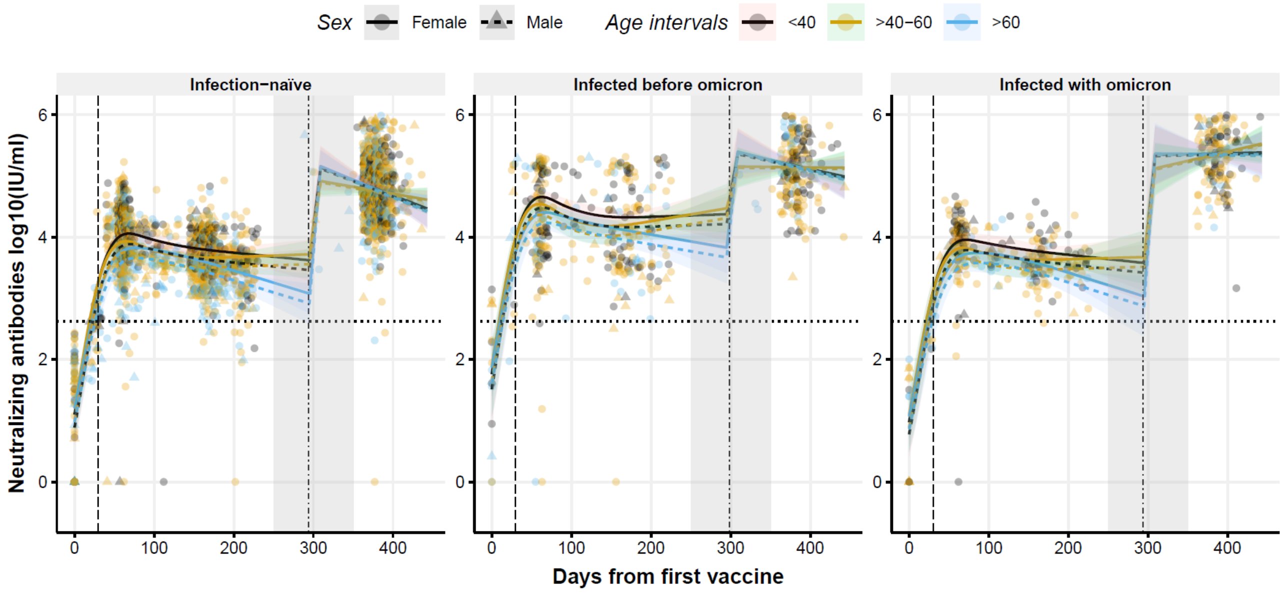

Supplementary Figure 2. Dynamics of circulating neutralizing antibody levels against RBD after the first dose of the BNT162b2 vaccine using two-part independent models. Distribution of neutralizing antibody levels, represented in log10(IU/ml), over time (days from the first vaccine) in infection-naïve individuals (left), in individuals previously infected with a variant before Omicron (middle), and in individuals infected with Omicron (right). Circles and triangles represent the observed levels of circulating neutralizing antibody in females and males, respectively. Solid and dashed lines represent the predicted levels of circulating neutralizing antibody calculated by the model in females and males, respectively. Black, yellow, and blue colors represent individuals with age <40, 40–60, and >60 years, respectively. Horizontal black dotted line represents the threshold for assay positivity. Vertical dashed and dashed-dotted lines represent when the second and the third dose was administered, respectively (median days). Shadowed areas represent the 95% confidence interval. Centre for the confidence interval is the predicted (mean) values. Predictive values in the grey-shaded area (days 250–350) do not represent a realistic projection due to insufficient observed data to provide realistic predictive data. Source data are provided as a Source Data file.

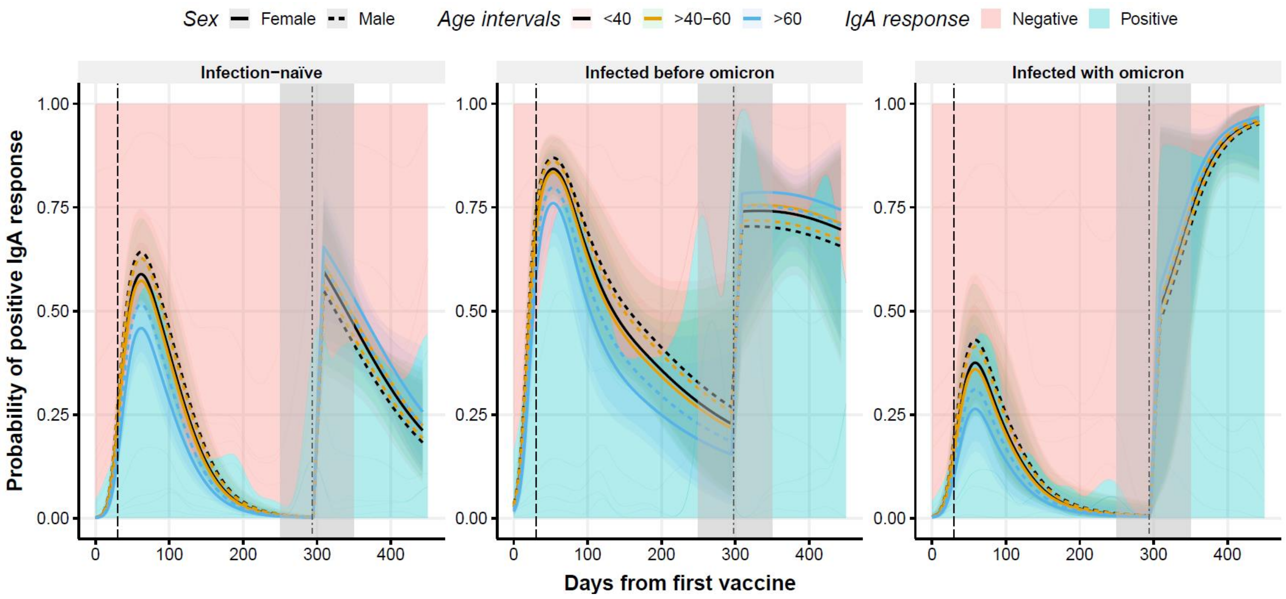

Supplementary Figure 3. Observed and predicted probability of positive IgA responses against RBD after the first dose of the BNT162b2 vaccine using two-part independent models. Distribution of positive IgA response (probability) over time (days from the first vaccine) in infection-naïve individuals (left), in individuals previously infected with a variant before Omicron (middle), and in individuals infected with Omicron (right). Blue and pink backgrounds represent the conditional density estimation of positive and negative IgA responses, respectively. Solid and dashed lines represent the predicted probability of positive IgA responses calculated by the model in females and males, respectively. Black, yellow, and blue colors represent individuals with age <40, 40–60, and >60 years, respectively. Vertical dashed and dashed-dotted lines represent when the second and the third dose was administered, respectively (median days). Shadowed areas represent the 95% confidence interval. Centre for the confidence interval is the predicted (mean) values. Predictive values in the grey-shaded area (days 250–350) do not represent a realistic projection due to insufficient observed data to provide realistic predictive data. Source data are provided as a Source Data file.

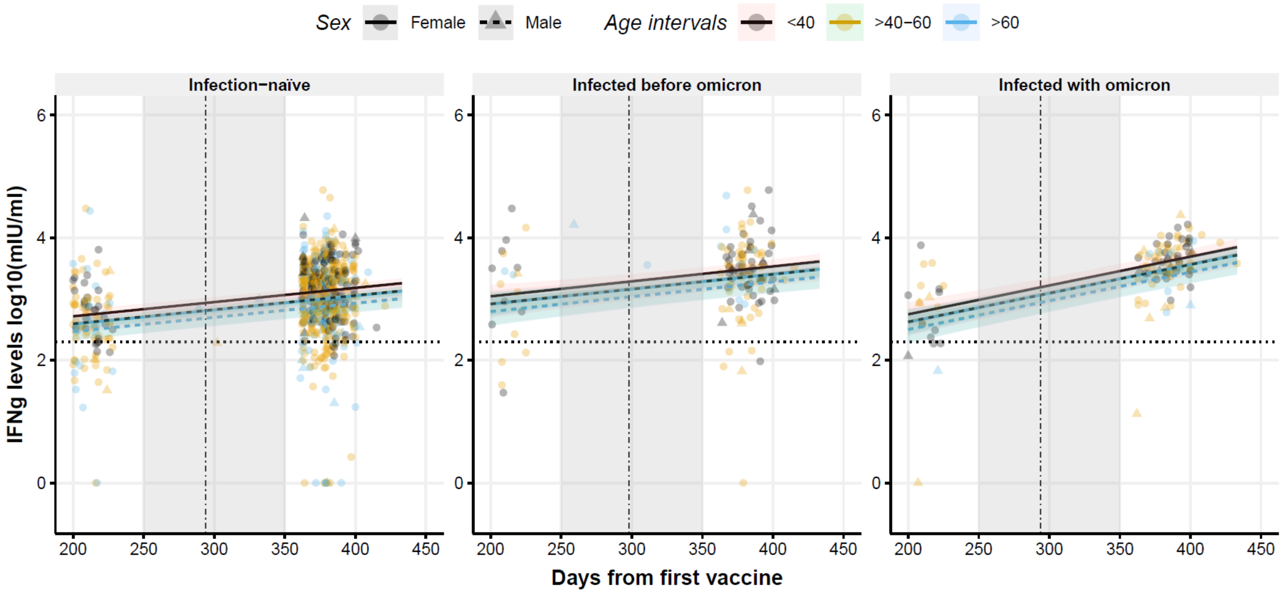

Supplementary Figure 4. Dynamics of IFN-γ levels released after the first dose of the BNT162b2 vaccine using a linear model. Distribution of IFN-γ levels, represented in log10(mIU/ml), over time (days from the first vaccine) in infection-naïve individuals (left), individuals infected with a variant before omicron (middle), and in individuals infected with Omicron (right). Circles and triangles represent the observed levels of IFN-γ levels released in females and males, respectively. Solid and dashed lines represent the predicted levels of IFN-γ levels released calculated by the model in females and males, respectively. Black, yellow, and blue colors represent individuals with age <40, 40–60, and >60 years, respectively. Horizontal black dotted line represents the threshold for assay positivity. Vertical dashed-dotted line represents when the third dose was administered (median days). Shadowed areas represent the 95% confidence interval. Centre for the confidence interval is the predicted (mean) values. Predictive values in the grey-shaded area (days 250–350) do not represent a realistic projection due to insufficient observed data to provide realistic predictive data. Source data are provided as a Source Data file.

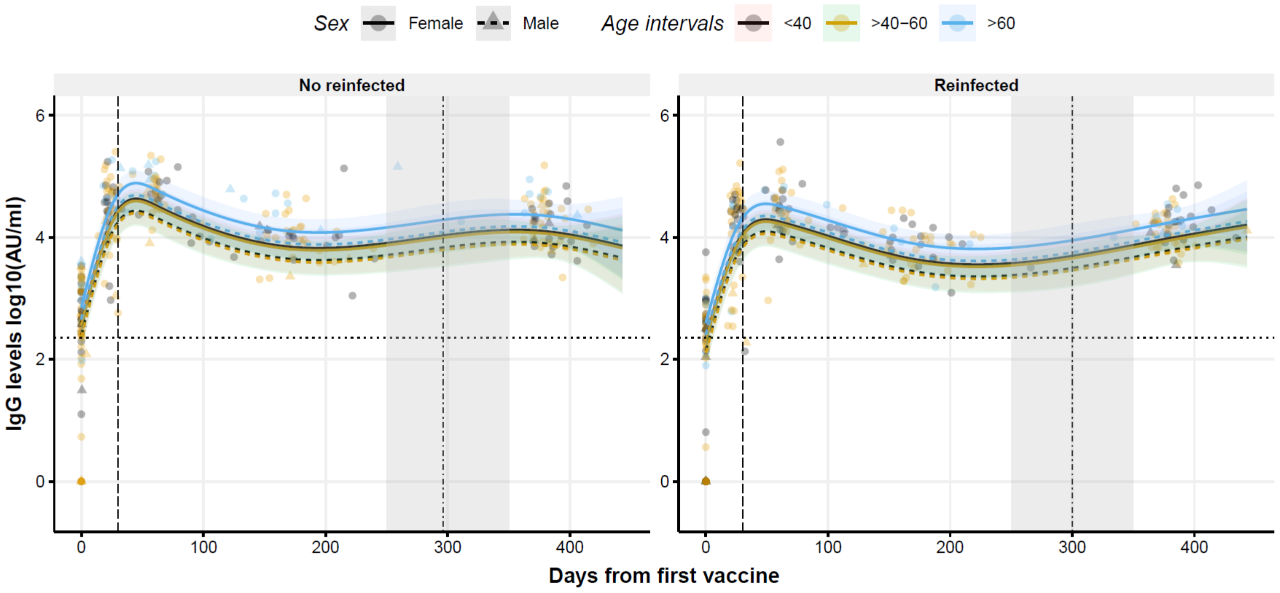

Supplementary Figure 5. Dynamics of circulating IgG levels against RBD after the first dose of the BNT162b2 vaccine in naturally infected individuals before Omicron in relation to reinfection using a non-linear model. Distribution of IgG levels, represented in log10(AU/ml), over time (days from the first vaccine) in individuals previously infected with a variant before Omicron without subsequent reinfection (left), and those who got a subsequent reinfection (right). Circles and triangles represent the observed levels of circulating IgG levels in females and males, respectively. Solid and dashed lines represent the predicted levels of circulating IgG levels calculated by the model in females and males, respectively. Black, yellow, and blue colors represent individuals with age <40, 40–60, and >60 years, respectively. Horizontal black dotted line represents the threshold for assay positivity. Vertical dashed and dashed-dotted lines represent when the second and the third dose was administered, respectively (median days). Shadowed areas represent the 95% confidence interval. Centre for the confidence interval is the predicted (mean) values. Predictive values in the grey-shaded area (days 250–350) do not represent a realistic projection due to insufficient observed data to provide realistic predictive data. Source data are provided as a Source Data file.

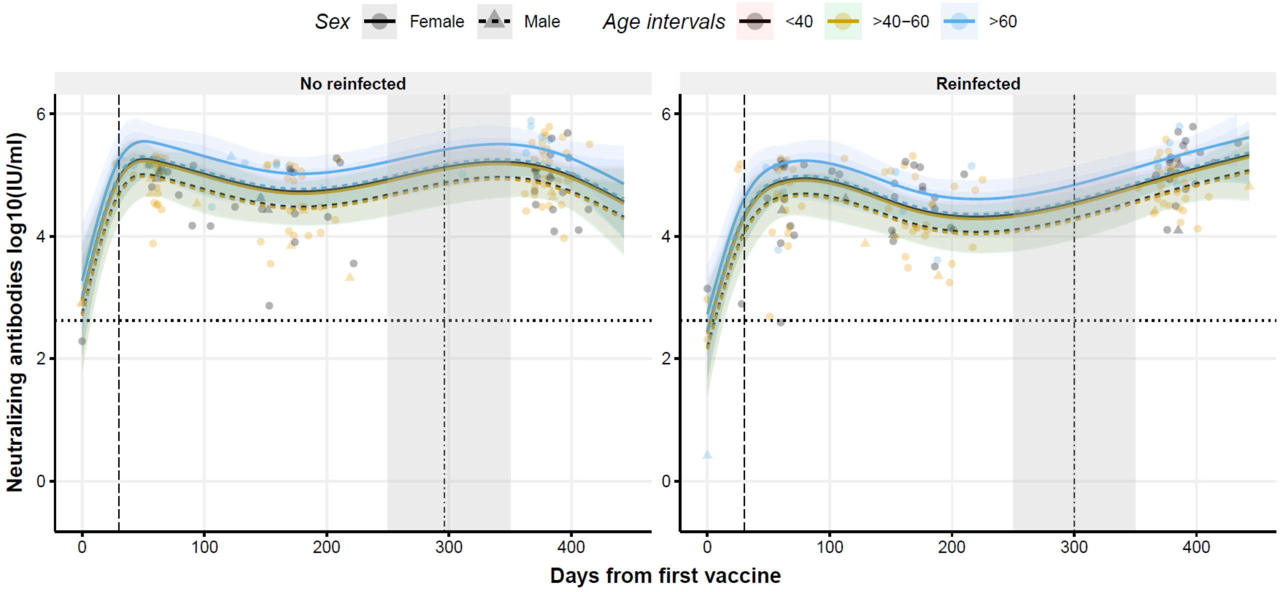

Supplementary Figure 6. Dynamics of circulating neutralizing antibody levels against RBD after the first dose of the BNT162b2 vaccine in naturally infected individuals before Omicron in relation to reinfection using a non-linear model. Distribution of neutralizing antibodies levels, represented in log10(IU/ml), over time (days from the first vaccine) in individuals previously infected with a variant before Omicron without subsequent reinfection (left), and those who got a subsequent reinfection (right). Circles and triangles represent the observed levels of circulating neutralizing antibodies in females and males, respectively. Solid and dashed lines represent the predicted levels of circulating neutralizing antibodies calculated by the model in females and males, respectively. Black, yellow, and blue colors represent individuals with age <40, 40–60, and >60 years, respectively. Horizontal black dotted line represents the threshold for assay positivity. Vertical dashed and dashed-dotted lines represent when the second and the third dose was administered, respectively (median days). Shadowed areas represent the 95% confidence interval. Centre for the confidence interval is the predicted (mean) values. Predictive values in the grey-shaded area (days 250–350) do not represent a realistic projection due to insufficient observed data to provide realistic predictive data. Source data are provided as a Source Data file.

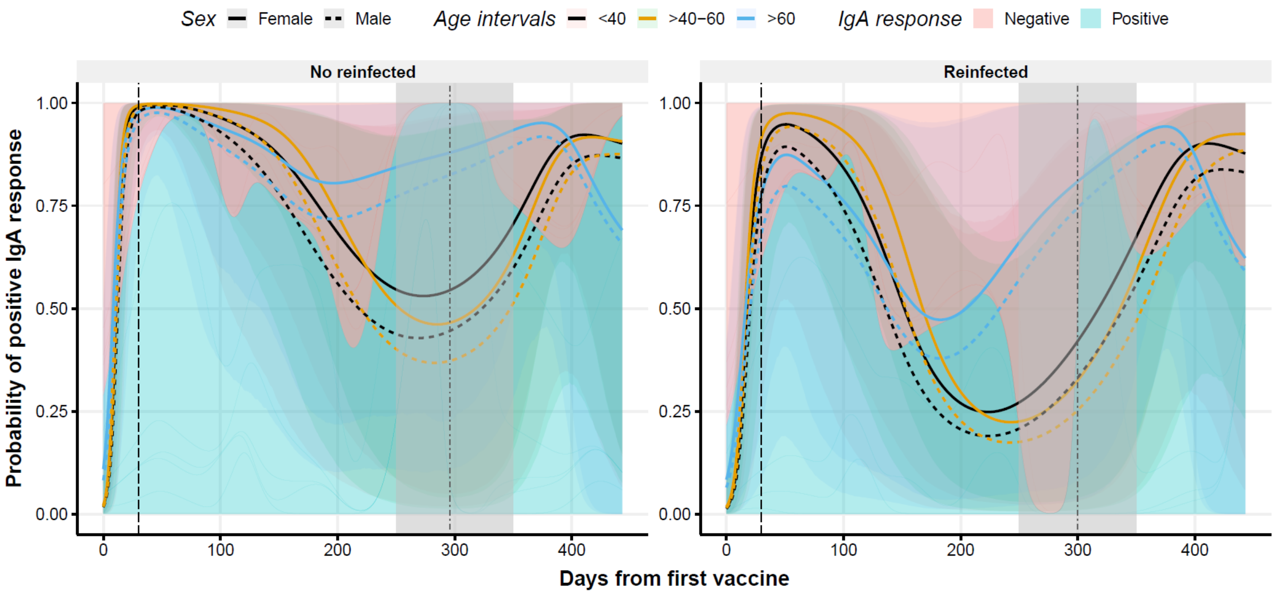

Supplementary Figure 7. Observed and predicted probability of positive IgA responses against RBD after the first dose of the BNT162b2 vaccine naturally infected individuals before Omicron in relation to reinfection using a non-linear model. Distribution of positive IgA response (probability) over time (days from the first vaccine) in individuals previously infected with a variant before Omicron without subsequent reinfection (left), and those who got a subsequent reinfection (right). Blue and pink backgrounds represent the conditional density estimation of positive and negative IgA responses, respectively. Solid and dashed lines represent the predicted probability of positive IgA responses calculated by the model in females and males, respectively. Vertical dashed and dashed-dotted lines represent when the second and the third dose was administered, respectively (median days). Shadowed areas represent the 95% confidence interval. Centre for the confidence interval is the predicted (mean) values. Predictive values in the grey-shaded area (days 250–350) do not represent a realistic projection due to insufficient observed data to provide realistic predictive data. Source data are provided as a Source Data file.

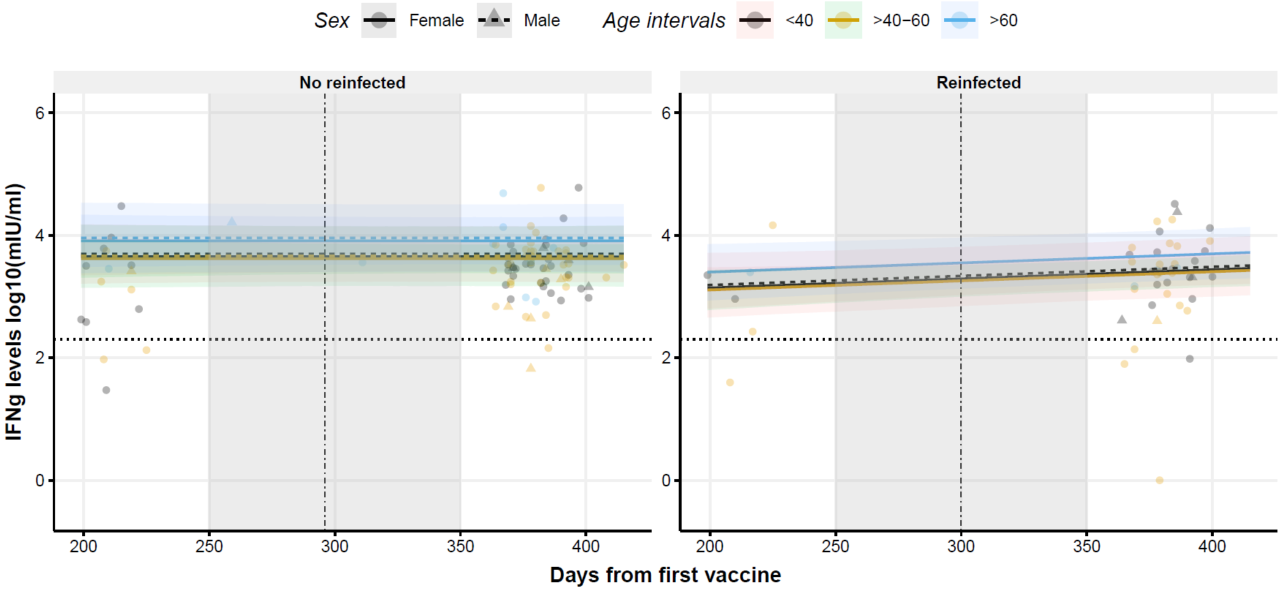

Supplementary Figure 8. Dynamics of circulating IFN-γ levels released after the first dose of the BNT162b2 vaccine in naturally infected individuals before Omicron in relation to reinfection using a linear model. Distribution of IFN-γ levels, represented in log10(AU/ml), over time (days from the first vaccine) in individuals previously infected with a variant before Omicron without subsequent reinfection (left), and those who got a subsequent reinfection (right). Circles and triangles represent the observed levels of IFN-γ levels released in females and males, respectively. Solid and dashed lines represent the predicted levels of IFN-γ levels released calculated by the model in females and males, respectively. Black, yellow, and blue colors represent individuals with age <40, 40–60, and >60 years, respectively. Horizontal black dotted line represents the threshold for assay positivity. Vertical dashed-dotted line represents when the third dose was administered (median days). Shadowed areas represent the 95% confidence interval. Centre for the confidence interval is the predicted (mean) values. Predictive values in the grey-shaded area (days 250–350) do not represent a realistic projection due to insufficient observed data to provide realistic predictive data. Source data are provided as a Source Data file.

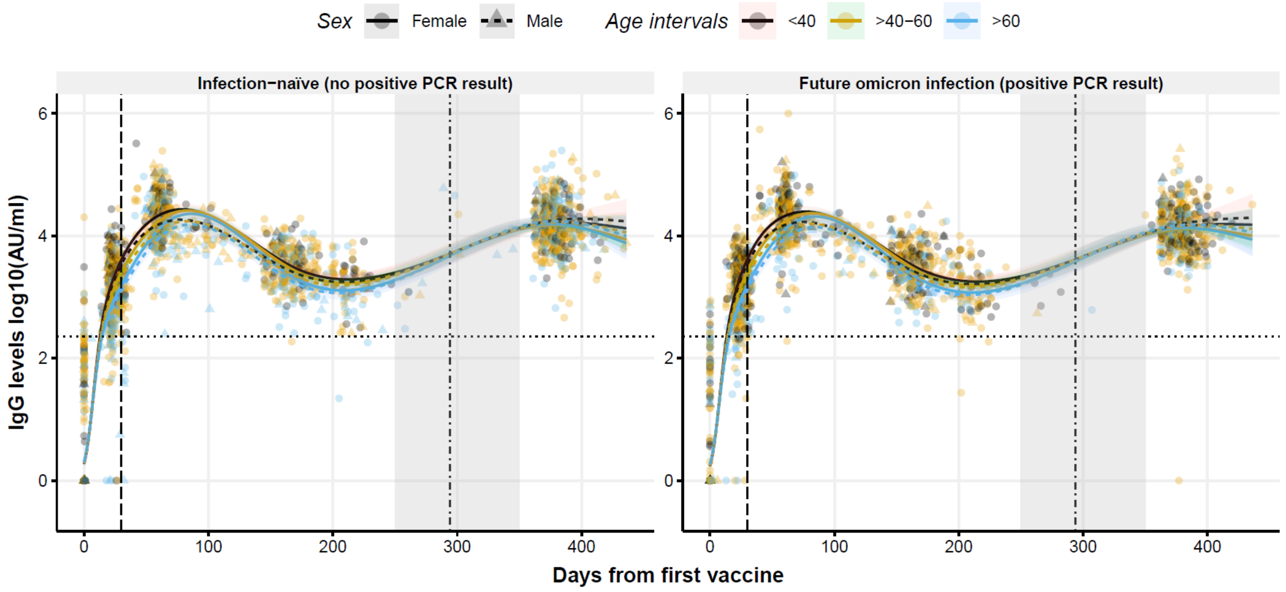

Supplementary Figure 9. Dynamics of circulating IgG levels against RBD after the first dose of the BNT162b2 vaccine in infection-naïve individuals in relation to future infection using a non-linear model. Distribution of IgG levels, represented in log10(AU/ml), over time (days from the first vaccine) in infection-naïve individuals who do not get a future infection (left), and those who get a future infection with Omicron (right). Circles and triangles represent the observed levels of circulating IgG levels in females and males, respectively. Solid and dashed lines represent the predicted levels of circulating IgG levels calculated by the model in females and males, respectively. Black, yellow, and blue colors represent individuals with age <40, 40–60, and >60 years, respectively. Horizontal black dotted line represents the threshold for assay positivity. Vertical dashed and dashed-dotted lines represent when the second and the third dose was administered, respectively (median days). Shadowed areas represent the 95% confidence interval. Centre for the confidence interval is the predicted (mean) values. Predictive values in the grey-shaded area (days 250–350) do not represent a realistic projection due to insufficient observed data to provide realistic predictive data. Source data are provided as a Source Data file.

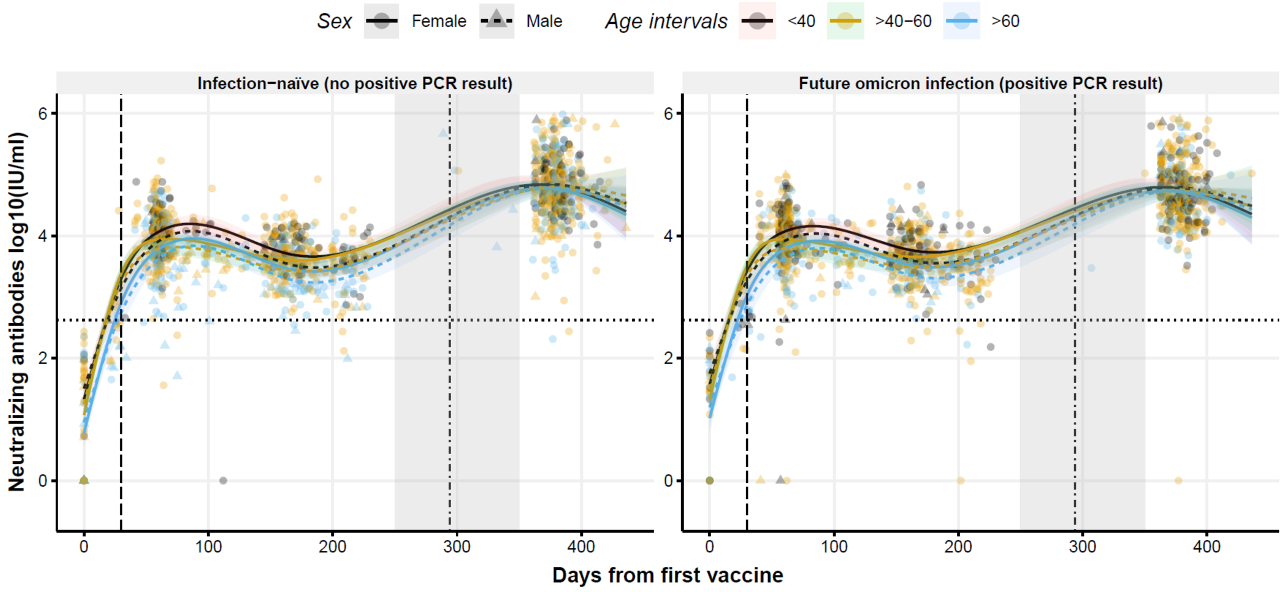

Supplementary Figure 10. Dynamics of circulating neutralizing antibody levels against RBD after the first dose of the BNT162b2 vaccine in infection-naïve individuals in relation to future infection using a non-linear model. Distribution of neutralizing antibodies levels, represented in log10(IU/ml), over time (days from the first vaccine) in infection-naïve individuals who do not get a future infection (left), and those who get a future infection with Omicron (right). Circles and triangles represent the observed levels of circulating neutralizing antibody levels in females and males, respectively. Solid and dashed lines represent the predicted levels of circulating neutralizing antibody levels calculated by the model in females and males, respectively. Black, yellow, and blue colors represent individuals with age <40, 40–60, and >60 years, respectively. Horizontal black dotted line represents the threshold for assay positivity. Vertical dashed and dashed-dotted lines represent when the second and the third dose was administered, respectively (median days). Shadowed areas represent the 95% confidence interval. Centre for the confidence interval is the predicted (mean) values. Predictive values in the grey-shaded area (days 250–350) do not represent a realistic projection due to insufficient observed data to provide realistic predictive data. Source data are provided as a Source Data file.

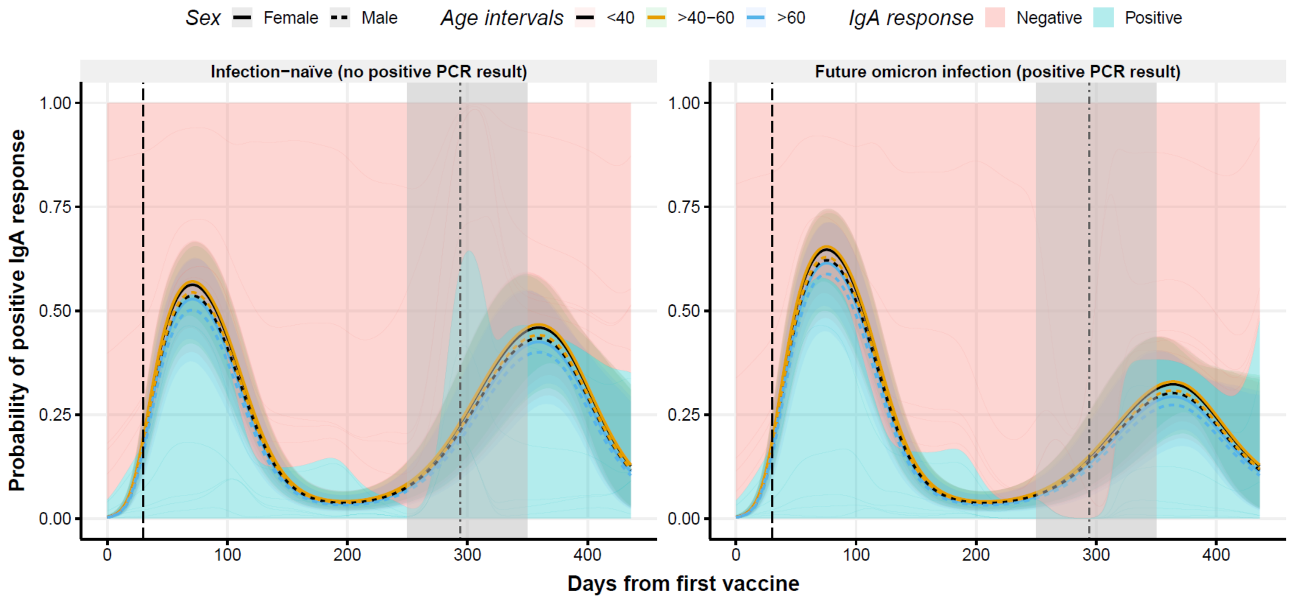

Supplementary Figure 11. Observed and predicted probability of positive IgA responses against RBD after the first dose of the BNT162b2 vaccine in infection-naïve individuals in relation to future infection using a non-linear model. Distribution of positive IgA response (probability) over time (days from the first vaccine) in infection-naïve individuals who do not get a future infection (left), and those who get a future infection with Omicron (right). Blue and pink backgrounds represent the conditional density estimation of positive and negative IgA responses, respectively. Solid and dashed lines represent the predicted probability of positive IgA responses calculated by the model in females and males, respectively. Black, yellow, and blue colors represent individuals with age <40, 40–60, and >60 years, respectively. Vertical dashed and dashed-dotted lines represent when the second and the third dose was administered, respectively (median days). Shadowed areas represent the 95% confidence interval. Centre for the confidence interval is the predicted (mean) values. Predictive values in the grey-shaded area (days 250–350) do not represent a realistic projection due to insufficient observed data to provide realistic predictive data. Source data are provided as a Source Data file.

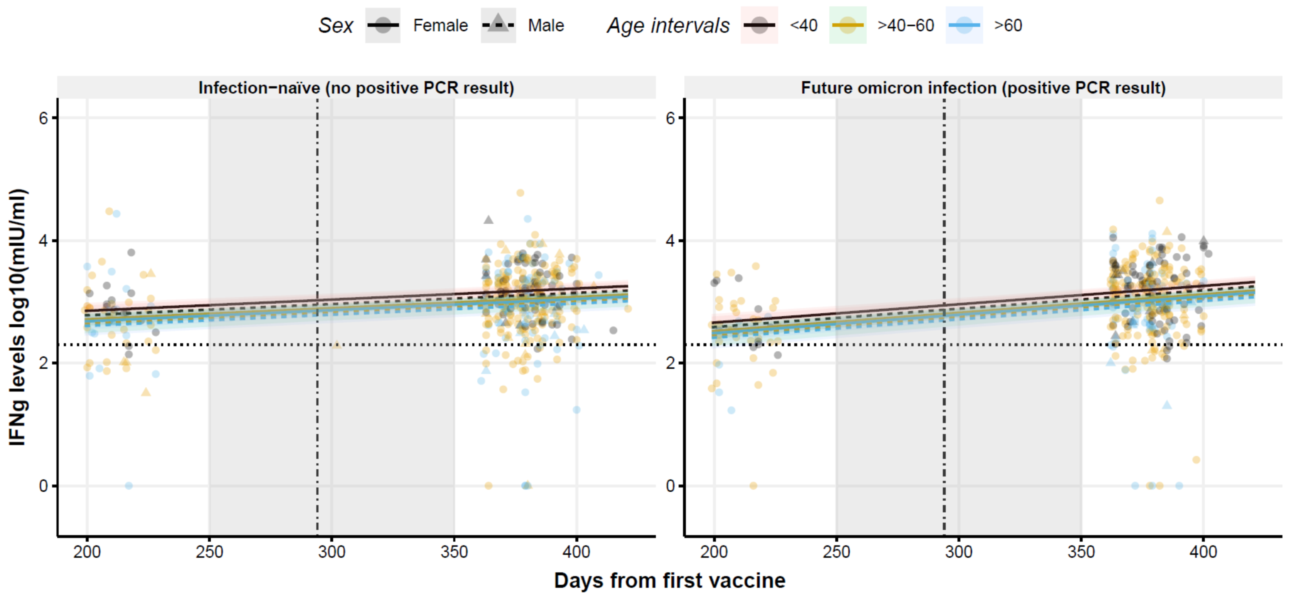

Supplementary Figure 12. Dynamics of IFN-γ levels released after the first dose of the BNT162b2 vaccine in infection-naïve individuals in relation to future infection using a linear model. Distribution of IFN-γ levels, represented in log10(mIU/ml), over time (days from the first vaccine) in infection-naïve individuals who do not get a future infection (left), and those who get a future infection with Omicron (right). Circles and triangles represent the observed levels of IFN-γ levels released in females and males, respectively. Solid and dashed lines represent the predicted levels of IFN-γ levels released calculated by the model in females and males, respectively. Black, yellow, and blue colors represent individuals with age <40, 40–60, and >60 years, respectively. Horizontal black dotted line represents the threshold for assay positivity. Vertical dashed-dotted line represents when the third dose was administered (median days). Shadowed areas represent the 95% confidence interval. Centre for the confidence interval is the predicted (mean) values. Predictive values in the grey-shaded area (days 250–350) do not represent a realistic projection due to insufficient observed data to provide realistic predictive data. Source data are provided as a Source Data file.

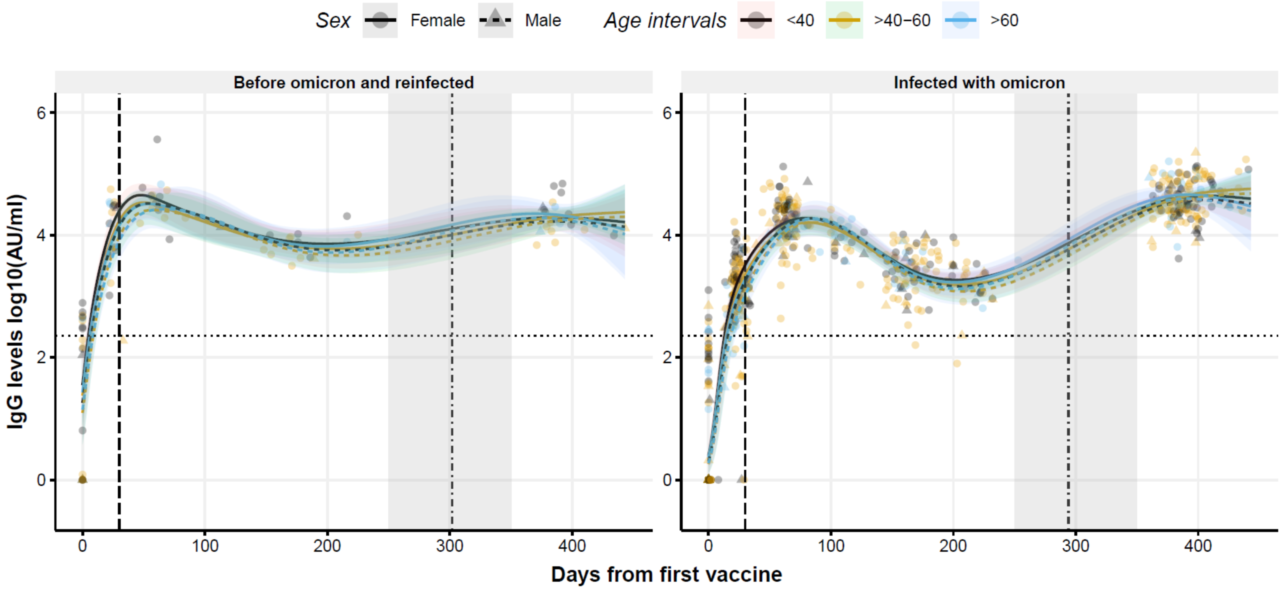

Supplementary Figure 13. Dynamics of circulating IgG levels against RBD after the first dose of the BNT162b2 vaccine in infected individuals with different immune imprinting status using a non-linear model. Distribution of IgG levels, represented in log10(AU/ml), over time (days from the first vaccine) in individuals previously infected before Omicron who got reinfected (left), and individuals infected with Omicron (right). Circles and triangles represent the observed levels of circulating IgG levels in females and males, respectively. Solid and dashed lines represent the predicted levels of circulating IgG levels calculated by the model in females and males, respectively. Black, yellow, and blue colors represent individuals with age <40, 40–60, and >60 years, respectively. Horizontal black dotted line represents the threshold for assay positivity. Vertical dashed and dashed-dotted lines represent when the second and the third dose was administered, respectively (median days). Shadowed areas represent the 95% confidence interval. Centre for the confidence interval is the predicted (mean) values. Predictive values in the grey-shaded area (days 250–350) do not represent a realistic projection due to insufficient observed data to provide realistic predictive data. Source data are provided as a Source Data file.

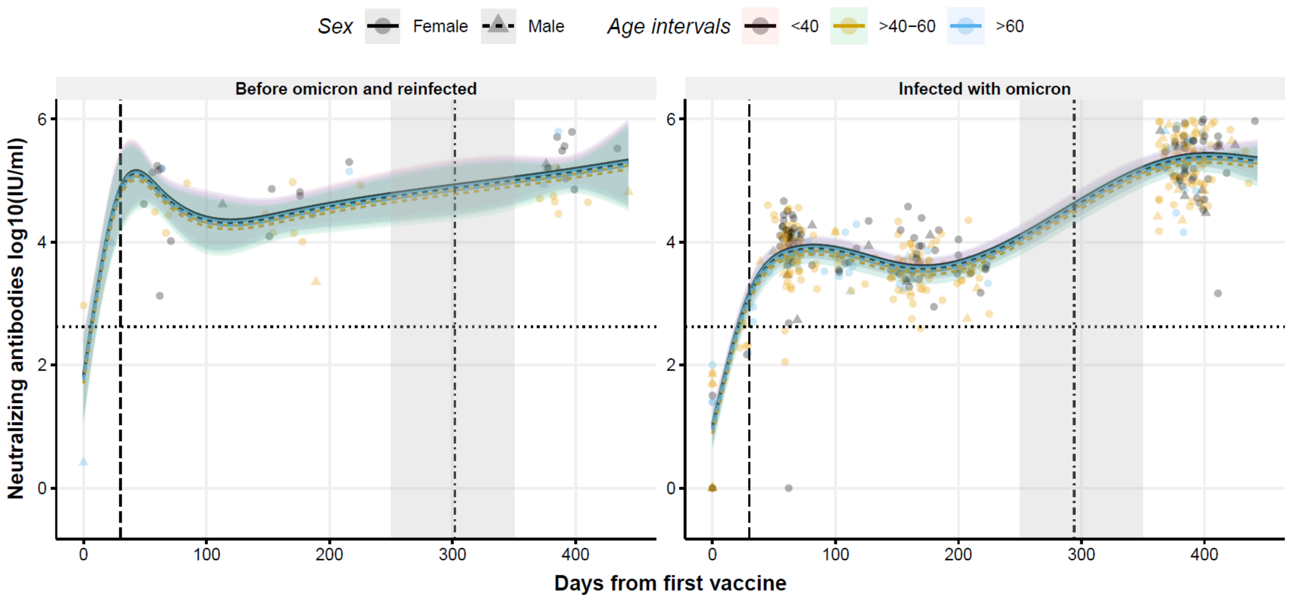

Supplementary Figure 14. Dynamics of circulating neutralizing antibody levels against RBD after the first dose of the BNT162b2 vaccine in individuals with different immune imprinting status using a non-linear model. Distribution of neutralizing antibody levels, represented in log10(IU/ml), over time (days from the first vaccine) in individuals previously infected before Omicron who got reinfected (left), and individuals infected with Omicron (right). Circles and triangles represent the observed levels of circulating neutralizing antibody levels in females and males, respectively. Solid and dashed lines represent the predicted levels of circulating neutralizing antibody levels calculated by the model in females and males, respectively. Black, yellow, and blue colors represent individuals with age <40, 40–60, and >60 years, respectively. Horizontal black dotted line represents the threshold for assay positivity. Vertical dashed and dashed-dotted lines represent when the second and the third dose was administered, respectively (median days). Shadowed areas represent the 95% confidence interval. Centre for the confidence interval is the predicted (mean) values. Predictive values in the grey-shaded area (days 250–350) do not represent a realistic projection due to insufficient observed data to provide realistic predictive data. Source data are provided as a Source Data file.

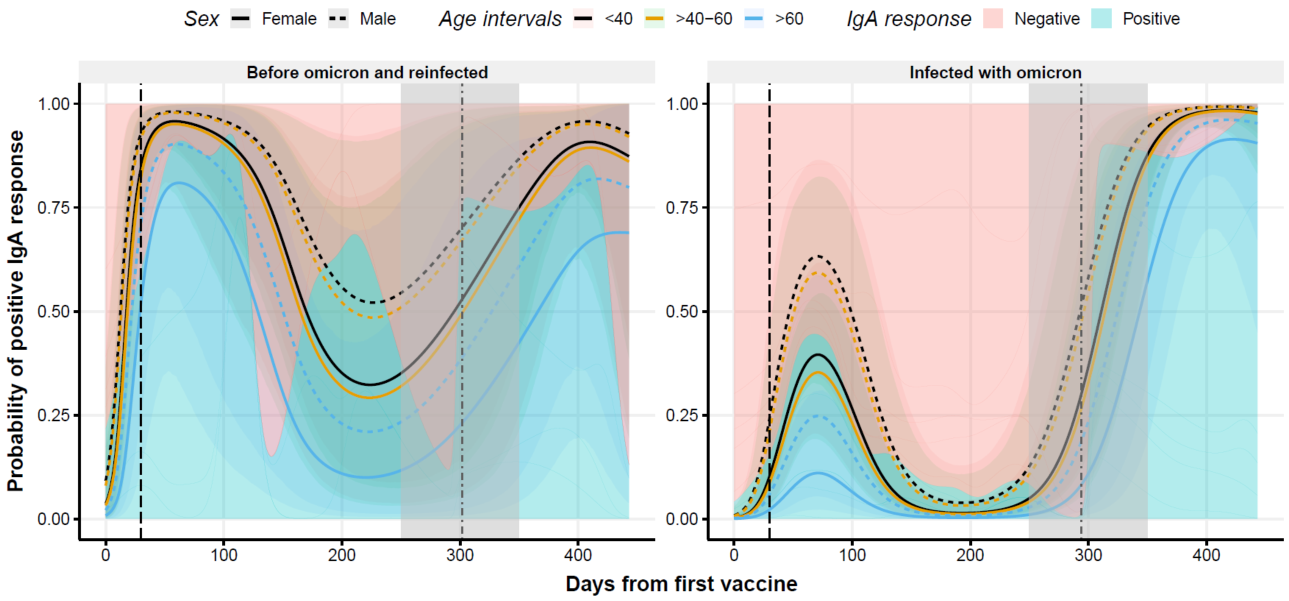

Supplementary Figure 15. Observed and predicted probability of positive IgA responses against RBD after the first dose of the BNT162b2 vaccine in individuals with different immune imprinting status using a non-linear model. Distribution of IgA responses (probability), over time (days from the first vaccine) in individuals infected before Omicron who got reinfected (left), and individuals infected with Omicron (right). Blue and pink backgrounds represent the conditional density estimation of positive and negative IgA responses, respectively. Solid and dashed lines represent the predicted probability of positive IgA responses calculated by the model in females and males, respectively. Black, yellow, and blue colors represent individuals with age <40, 40–60, and >60 years, respectively. Vertical dashed and dashed-dotted lines represent when the second and the third dose was administered, respectively (median days). Shadowed areas represent the 95% confidence interval. Centre for the confidence interval is the predicted (mean) values. Predictive values in the grey-shaded area (days 250–350) do not represent a realistic projection due to insufficient observed data to provide realistic predictive data. Source data are provided as a Source Data file.

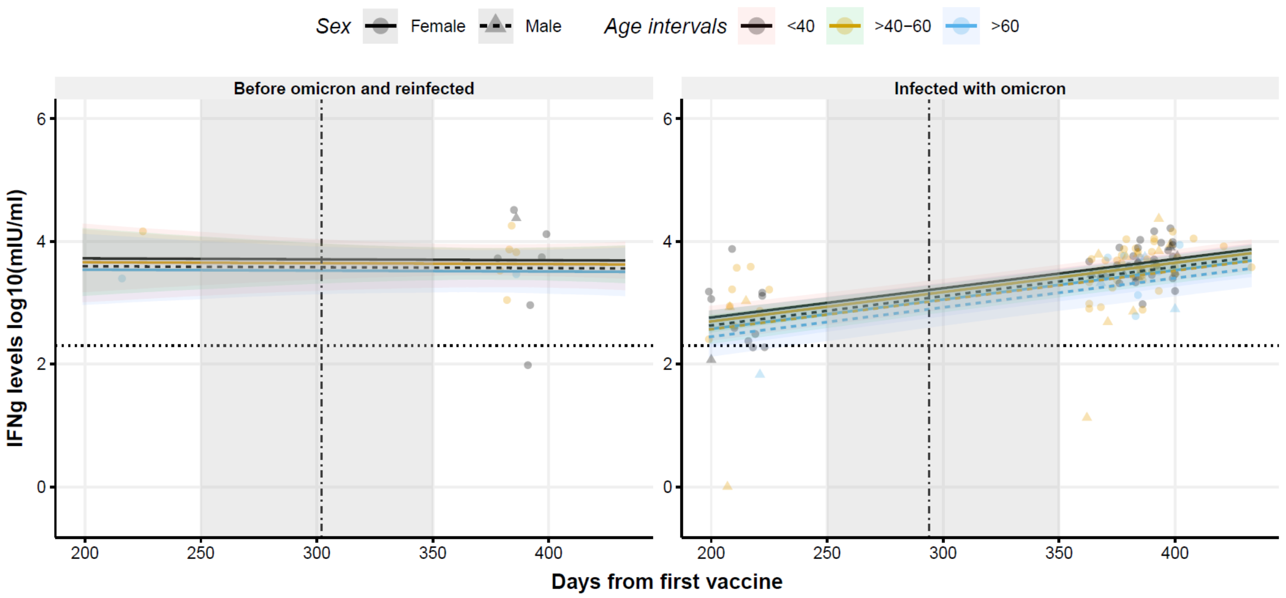

Supplementary Figure 16. Dynamics of IFN-γ levels released after the first dose of the BNT162b2 vaccine in individuals infected with different immune imprinting status using a linear model. Distribution of IFN-γ levels released, represented in log10(mIU/ml), over time (days from the first vaccine) in individuals infected before Omicron who got reinfected (left), and individuals infected with Omicron (right). Circles and triangles represent the observed levels of IFN-γ levels released in females and males, respectively. Solid and dashed lines represent the predicted levels of IFN-γ levels released calculated by the model in females and males, respectively. Black, yellow, and blue colors represent individuals with age <40, 40–60, and >60 years, respectively. Horizontal black dotted line represents the threshold for assay positivity. Vertical dashed-dotted line represents when the third dose was administered (median days). Shadowed areas represent the 95% confidence interval. Centre for the confidence interval is the predicted (mean) values. Predictive values in the grey-shaded area (days 250–350) do not represent a realistic projection due to insufficient observed data to provide realistic predictive data. Source Data are provided as a Source Data file.

Supplementary Tables

Supplementary Table 1. Demographic data and characteristics of the subgroup study cohorts

	Reinfection cohort (N=119)		Future infection cohort (N=955) *		Immune imprinting cohort (N=185)	
	**Not reinfected (N=62)**	**Reinfected (N=57)**	**Not future infected (N=492)**	**Future infected (N=463)**	**Before omicron (N=22)**	**Omicron (N=163)**
**Sex**						
Female	53 (85.5%)	51 (89.5%)	419 (85.2%)	414 (89.4%)	18 (81.8%)	132 (81.0%)
Male	9 (14.5%)	6 (10.5%)	73 (14.8%)	49 (10.6%)	4 (18.2%)	31 (19.0%)
**Age (years)**						
Median (IQR)	48 (33–55)	45 (35–56)	55 (46–61)	51 (41–59)	42 (35–49)	46 (39–55)
<40	19 (30.6%)	21 (36.8%)	81 (16.5%)	112 (24.2%)	11 (50.0%)	53 (32.5%)
>40–60	35 (56.5%)	30 (52.6%)	267 (54.3%)	251 (54.2%)	8 (36.4%)	93 (57.1%)
>60	8 (12.9%)	6 (10.5%)	144 (29.3%)	100 (21.6%)	3 (13.6%)	17 (10.4%)
**BMI**						
Median (IQR)	25 (22–29) [a]	24 (22–25) [b]	24 (22–28) [c]	24 (22–27) [d]	23 (21–25) [e]	24 (22–26) [f]
Underweight	0 (0%)	2 (3.8%)	8 (1.8%)	2 (0.5%)	0 (0%)	1 (0.8%)
Normal	30 (49.2%)	36 (67.9%)	235 (53.9%)	245 (58.8%)	13 (72.2%)	86 (64.7%)
Overweight	20 (32.8%)	11 (20.8%)	122 (28.0%)	105 (25.2%)	4 (22.2%)	35 (26.3%)
Obese	11 (18.0%)	4 (7.5%)	71 (16.3%)	65 (15.6%)	1 (5.6%)	11 (8.3%)
**Time between first and second dose (days)**						
Median (IQR)	30 (28–33)	30 (29–33)	30 (29–32)	30 (29–33)	30 (28–33)	30 (29–33)
**Time between first and third dose (days)**						
Median (IQR)	296 (287–302)	300 (294–310)	294 (286–302)	294 (287–301)	302 (296–325)	294 (285–302)
**Time between second dose and Omicron infection (days)**						
Median (IQR)	N.A.	N.A.	N.A.	N.A.	338 (324–348) [g]	337 (324–348) [g]

	Reinfection cohort (N=119)		Future infection cohort (N=955) *		Immune imprinting cohort (N=185)	
	**Not reinfected (N=62)**	**Reinfected (N=57)**	**Not future infected (N=492)**	**Future infected (N=463)**	**Before omicron (N=22)**	**Omicron (N=163)**
**Time between third dose and Omicron infection (days)**						
Median (IQR)	N.A.	N.A.	N.A.	N.A.	64 (49–80) [h]	74 (64–84) [h]
**Time from sample to Omicron infection (days)**						
Median (IQR)	N.A.	N.A.	N.A.	N.A.	18 (10–24) [i]	20 (14–27) [i]

*Future infection based on a positive RT-PCR result after the 12-month sampling round

[a] Missing value: 1 individual. [b] Missing values: 4 individuals. [c] Missing values: 56 individuals. [d] Missing values: 46 individuals. [e] Missing values: 4 individuals. [f] Missing values: 30 individuals

[g] p=0.8138 (Mann-Whitney test, two-sided). [h] p=0.0889 (Mann-Whitney test, two-sided). [i] p=0.3331 (Mann-Whitney test, two-sided). $p<0.05$ was considered significant.

IQR: Interquartile range

N.A.: Not applicable

Supplementary Table 2. Predicted mean IgG levels, represented in AU/ml, in individuals after the administration first vaccination dose according to infection status, age group, and sex

Days from 1st vaccine [a]	Infection status	Age Group	Sex	Geometric mean	CI 2.5%	CI 97.5%
79	Infection-naïve	<40	Female	26107.3	21798.5	31207.7
60	Infected before omicron	<40	Female	28684.5	22942.8	35984.4
79	Infected with omicron	<40	Female	19354.5	14565.6	25611.7
389	Infection-naïve	<40	Female	14999.2	13163.1	17131.0
378	Infected before omicron	<40	Female	23820.9	19433.3	29240.6
380*	Infected with omicron	<40	Female	38648.3	30394.3	48714.2
83	Infection-naïve	>40-60	Female	23926.1	21211.8	27015.4
70	Infected before omicron	>40-60	Female	23292.5	18863.1	28772.3
83	Infected with omicron	>40-60	Female	18665.5	14587.3	23910.6
387	Infection-naïve	>40-60	Female	14094.5	12961.0	15345.1
377	Infected before omicron	>40-60	Female	21710.4	18343.2	25733.0
425	Infected with omicron	>40-60	Female	46531.2	32647.9	66143.2
85	Infection-naïve	>60	Female	20934.7	17619.5	24898.7
77	Infected before omicron	>60	Female	33438.1	23645.3	47307.1
85	Infected with omicron	>60	Female	18532.9	12273.9	28098.7
373	Infection-naïve	>60	Female	14982.1	13302.1	16886.8
364	Infected before omicron	>60	Female	41707.6	30682.8	57098.5
388	Infected with omicron	>60	Female	46695.2	32277.5	67879.6
79	Infection-naïve	<40	Male	21008.6	16022.0	27441.0
60	Infected before omicron	<40	Male	15154.4	10455.7	21982.8
80	Infected with omicron	<40	Male	13082.2	8826.4	19413.1
391	Infection-naïve	<40	Male	16547.4	13413.2	20496.0
381	Infected before omicron	<40	Male	17201.6	12312.3	23958.5
380*	Infected with omicron	<40	Male	35519.9	25033.8	49900.3
83	Infection-naïve	>40-60	Male	19277.0	15183.0	24321.6
70	Infected before omicron	>40-60	Male	12340.9	8461.4	18072.5
83	Infected with omicron	>40-60	Male	12631.4	8768.1	18414.4
387	Infection-naïve	>40-60	Male	15511.8	13124.7	18376.3

380	Infected before omicron	>40-60	Male	15661.8	11361.6	21525.0
431	Infected with omicron	>40-60	Male	44169.1	24248.5	80014.7
85	Infection-naïve	>60	Male	16877.4	13069.0	21830.3
77	Infected before omicron	>60	Male	17752.7	11198.5	27798.5
86	Infected with omicron	>60	Male	12549.5	7669.8	20211.5
374	Infection-naïve	>60	Male	16299.5	13544.3	19570.3
367	Infected before omicron	>60	Male	29720.6	20005.4	44112.5
390	Infected with omicron	>60	Male	43221.5	27876.8	66880.0

[a] The day from 1st vaccine is selected based on when the peak after the second dose and the third dose was reached according to the infection status, age group, and sex

* IgG peak after the third dose did not plateau. Instead, the median days from the 12-months sampling round was selected.

CI: Confidence of interval

Source data are provided as a Source Data file

Supplementary Table 3. Predicted mean neutralizing antibody levels, represented in IU/ml, in individuals after the administration first vaccination dose according to infection status, age group, and sex

Days from 1st vaccine [a]	Infection status	Age Group	Sex	Geometric mean	CI 2.5%	CI 97.5%
89	Infection-naïve	<40	Female	14323.4	11010.7	18601.3
75	Infected before omicron	<40	Female	40540.9	31088.3	52708.3
91	Infected with omicron	<40	Female	11596.5	8229.9	16402.0
366	Infection-naïve	<40	Female	66250.7	53613.6	81675.6
386	Infected before omicron	<40	Female	146510.9	119373.0	180276.2
389	Infected with omicron	<40	Female	241503.0	193067.3	302896.8
80	Infection-naïve	>40-60	Female	8570.5	7303.6	10038.6
53	Infected before omicron	>40-60	Female	31407.6	24249.1	40645.2
84	Infected with omicron	>40-60	Female	6833.5	5118.1	9127.3
380	Infection-naïve	>40-60	Female	59410.0	53527.7	65841.5
380*	Infected before omicron	>40-60	Female	137566.1	114249.3	166039.4
380	Infected with omicron	>40-60	Female	223309.6	177122.6	281124.0
91	Infection-naïve	>60	Female	9220.4	7176.4	11792.7
81	Infected before omicron	>60	Female	25377.3	18278.8	35454.7
92	Infected with omicron	>60	Female	7483.7	5176.6	10771.0
365	Infection-naïve	>60	Female	66785.4	54835.1	81428.7
378	Infected before omicron	>60	Female	144622.0	115167.1	182531.2
384	Infected with omicron	>60	Female	235739.8	180479.9	307463.4
84	Infection-naïve	<40	Male	9508.3	6647.0	13653.2
64	Infected before omicron	<40	Male	29014.6	21406.8	39330.1
87	Infected with omicron	<40	Male	7638.5	4980.0	11715.2
363	Infection-naïve	<40	Male	66500.5	48285.1	91530.6
377	Infected before omicron	<40	Male	142631.1	108033.0	188342.4
383	Infected with omicron	<40	Male	231774.4	175080.1	308191.0
71	Infection-naïve	>40-60	Male	5956.2	4652.0	7671.7
51	Infected before omicron	>40-60	Male	24860.5	16899.9	36185.3
76	Infected with omicron	>40-60	Male	4654.5	3352.5	6529.4
374	Infection-naïve	>40-60	Male	58324.4	46623.3	73005.6

380*	Infected before omicron	>40-60	Male	134217.5	103462.1	173850.3
408	Infected with omicron	>40-60	Male	243767.4	155162.1	383631.1
87	Infection-naïve	>60	Male	6074.7	4340.2	8382.9
73	Infected before omicron	>60	Male	17498.8	12260.8	24714.5
89	Infected with omicron	>60	Male	4900.9	3180.0	7456.8
363	Infection-naïve	>60	Male	67086.8	49747.9	90406.9
373	Infected before omicron	>60	Male	142498.1	105746.3	194067.1
380	Infected with omicron	>60	Male	228963.9	169009.9	312731.2

[a] The day from 1st vaccine is selected based on when the peak after the second dose and the third dose was reached according to the infection status, age group, and sex

* Neutralizing antibodies peak after the third dose did not plateau. Instead, the median days from the 12-months sampling round was selected.

CI: Confidence of interval

Source data are provided as a Source Data file

Supplementary Table 4. Predicted mean probability of positive IgA responses, represented in percentage, in individuals after the administration first vaccination dose according to infection status, age group, and sex

Days from 1st vaccine [a]	Infectious status	Age Group	Sex	Geometric mean	CI 2.5%	CI 97.5%
74	Infection-naïve	<40	Female	67.1	56.3	76.7
64	Infected before omicron	<40	Female	85.5	77.3	91.4
71	Infected with omicron	<40	Female	43.5	28.9	58.8
363	Infection-naïve	<40	Female	33.4	22.2	45.9
388	Infected before omicron	<40	Female	82.2	73.2	89.1
418	Infected with omicron	<40	Female	96.7	87.7	99.6
74	Infection-naïve	>40-60	Female	64.2	57.0	71.0
62	Infected before omicron	>40-60	Female	84.0	76.4	89.9
71	Infected with omicron	>40-60	Female	40.2	27.2	54.4
367	Infection-naïve	>40-60	Female	34.9	28.0	42.5
400	Infected before omicron	>40-60	Female	84.7	74.7	91.9
428	Infected with omicron	>40-60	Female	97.8	89.7	99.9
71	Infection-naïve	>60	Female	43.8	33.6	54.3
53	Infected before omicron	>60	Female	72.0	60.0	82.0
66	Infected with omicron	>60	Female	23.4	13.1	36.4
355	Infection-naïve	>60	Female	44.9	30.9	59.6
374	Infected before omicron	>60	Female	86.6	78.2	92.6
405	Infected with omicron	>60	Female	96.7	90.1	99.3
74	Infection-naïve	<40	Male	69.2	56.4	80.1
64	Infected before omicron	<40	Male	86.6	77.8	92.7
71	Infected with omicron	<40	Male	45.9	29.8	62.8
364	Infection-naïve	<40	Male	35.7	22.7	50.3
388	Infected before omicron	<40	Male	83.5	73.6	90.7
418	Infected with omicron	<40	Male	97.0	88.6	99.7
74	Infection-naïve	>40-60	Male	66.4	55.9	75.5
62	Infected before omicron	>40-60	Male	85.2	76.4	91.5
71	Infected with omicron	>40-60	Male	42.6	27.8	58.6
368	Infection-naïve	>40-60	Male	37.3	27.1	48.2

400	Infected before omicron	>40-60	Male	85.9	75.3	92.9
428	Infected with omicron	>40-60	Male	98.0	90.5	99.9
71	Infection-naïve	>60	Male	46.3	33.6	59.1
53	Infected before omicron	>60	Male	73.9	60.6	84.5
66	Infected with omicron	>60	Male	25.3	13.8	40.0
355	Infection-naïve	>60	Male	47.4	31.6	64.1
374	Infected before omicron	>60	Male	87.6	79.0	93.6
406	Infected with omicron	>60	Male	97.0	90.5	99.4

[a] The day from 1st vaccine is selected based on when the peak after the second dose and the third dose was reached according to the infection status, age group, and sex

CI: Confidence of interval

Source data are provided as a Source Data file

Supplementary Table 5. Predicted mean IFN-γ levels, represented in mIU/ml, in individuals after the administration first vaccination dose according to infection status, age group, and sex

Days from 1st vaccine [a]	Infectious status	Age Group	Sex	Geometric mean	CI 2.5%	CI 97.5%
210	Infection-naïve	<40	Female	551.9	438.1	689.7
210	Infected before omicron	<40	Female	1167.9	773.9	1771.3
210	Infected with omicron	<40	Female	624.0	408.8	961.9
380	Infection-naïve	<40	Female	1366.9	1147.6	1628.7
380	Infected before omicron	<40	Female	3041.2	2380.4	3866.9
380	Infected with omicron	<40	Female	3949.4	3007.7	5146.0
210	Infection-naïve	>40-60	Female	416.2	340.5	508.0
210	Infected before omicron	>40-60	Female	880.8	578.0	1325.8
210	Infected with omicron	>40-60	Female	470.6	309.4	716.9
380	Infection-naïve	>40-60	Female	1030.8	902.1	1175.7
380	Infected before omicron	>40-60	Female	2293.4	1808.4	2914.1
380	Infected with omicron	>40-60	Female	2978.4	2323.3	3826.8
210	Infection-naïve	>60	Female	410.9	323.6	523.8
210	Infected before omicron	>60	Female	869.4	559.2	1354.6
210	Infected with omicron	>60	Female	464.5	297.8	725.3
380	Infection-naïve	>60	Female	1017.6	844.5	1226.6
380	Infected before omicron	>60	Female	2263.9	1708.0	3009.7
380	Infected with omicron	>60	Female	2940.0	2180.5	3962.4
210	Infection-naïve	<40	Male	414.7	288.6	575.1
210	Infected before omicron	<40	Male	873.2	526.9	1416.8
210	Infected with omicron	<40	Male	468.5	285.7	754.4
380	Infection-naïve	<40	Male	1021.0	718.7	1381.1
380	Infected before omicron	<40	Male	2259.8	1502.2	3198.4
380	Infected with omicron	<40	Male	2929.7	1935.0	4185.4
210	Infection-naïve	>40-60	Male	313.3	222.7	427.3
210	Infected before omicron	>40-60	Male	659.8	395.8	1064.2
210	Infected with omicron	>40-60	Male	354.0	217.7	567.3
380	Infection-naïve	>40-60	Male	771.4	551.6	1016.9

380	Infected before omicron	>40-60	Male	1707.4	1134.3	2405.1
380	Infected with omicron	>40-60	Male	2213.5	1475.0	3120.6
210	Infection-naïve	>60	Male	309.3	215.4	428.3
210	Infected before omicron	>60	Male	651.3	387.7	1075.9
210	Infected with omicron	>60	Male	349.4	211.9	568.7
380	Infection-naïve	>60	Male	761.5	537.9	1027.2
380	Infected before omicron	>60	Male	1685.5	1100.4	2449.6
380	Infected with omicron	>60	Male	2185.1	1429.0	3165.8

[a] The day from 1st vaccine is selected based on the median of days from the first vaccine at 6- and 12-month rounds according to the infection status, age group, and sex

CI: Confidence of interval

Source data are provided as a Source Data file

Supplementary Table 6. Predicted mean IgG levels, represented in AU/ml, in individuals after the administration first vaccination dose according to reinfection status, age group, and sex

Days from 1st vaccine [a]	Reinfection status	Age Group	Sex	Geometric mean	CI 2.5%	CI 97.5%
49	Reinfected	<40	Female	19670.3	13693.8	27950.8
45	No reinfected	<40	Female	42971.3	29434.4	61865.0
180	Reinfected	<40	Female	4099.2	2805.4	6009.9
180	No reinfected	<40	Female	6783.3	4639.6	10005.2
380	Reinfected	<40	Female	9692.7	6675.3	13933.5
380	No reinfected	<40	Female	12584.9	8728.9	18061.7
49	Reinfected	>40-60	Female	18061.4	12945.1	25069.3
45	No reinfected	>40-60	Female	39456.7	28123.8	54540.4
180	Reinfected	>40-60	Female	3763.9	2630.3	5337.5
180	No reinfected	>40-60	Female	6228.5	4383.0	8863.4
380	Reinfected	>40-60	Female	8899.9	6297.4	12378.7
380	No reinfected	>40-60	Female	11555.6	8289.6	16014.8
49	Reinfected	>60	Female	35661.7	22114.7	57838.5
45	No reinfected	>60	Female	77905.9	47798.4	125488.4
180	Reinfected	>60	Female	7431.7	4537.2	12309.1
180	No reinfected	>60	Female	12298.0	7471.0	20022.9
380	Reinfected	>60	Female	17572.6	10762.2	28422.8
380	No reinfected	>60	Female	22816.1	13934.5	37189.7
49	Reinfected	<40	Male	12404.9	7565.1	20304.0
45	No reinfected	<40	Male	27099.6	16571.8	44603.3
180	Reinfected	<40	Male	2585.1	1547.1	4333.5
180	No reinfected	<40	Male	4277.9	2589.0	7186.0
380	Reinfected	<40	Male	6112.6	3722.1	10152.7
380	No reinfected	<40	Male	7936.6	4858.0	12970.9
49	Reinfected	>40-60	Male	11390.3	7018.1	18370.7
45	No reinfected	>40-60	Male	24883.1	15519.3	39842.7
180	Reinfected	>40-60	Male	2373.7	1418.6	3922.7
180	No reinfected	>40-60	Male	3928.0	2371.4	6409.4

380	Reinfected	>40-60	Male	5612.7	3414.2	9146.8
380	No reinfected	>40-60	Male	7287.4	4512.0	11746.3
49	Reinfected	>60	Male	22489.8	12753.0	39385.3
45	No reinfected	>60	Male	49130.8	28204.4	85464.5
180	Reinfected	>60	Male	4686.8	2623.0	8426.4
180	No reinfected	>60	Male	7755.6	4340.0	13659.0
380	Reinfected	>60	Male	11082.0	6246.5	19648.6
380	No reinfected	>60	Male	14388.8	8258.3	25183.3

[a] The day from 1st vaccine is selected based on the IgG peak after second dose of vaccination, waning period and at the median days from first vaccine dose at the 12-month round (as the IgG levels plateaued) according to the reinfection status, age group, and sex

CI: Confidence of interval

Source data are provided as a Source Data file

Supplementary Table 7. Predicted mean neutralizing antibody levels, represented in IU/ml, in individuals after the administration first vaccination dose according to reinfection status, age group, and sex

Days from 1st vaccine [a]	Reinfection status	Age Group	Sex	Geometric mean	CI 2.5%	CI 97.5%
80	Reinfected	<40	Female	87550.6	47314.0	164557.3
51	No reinfected	<40	Female	181644.7	91917.4	359418.3
180	Reinfected	<40	Female	25015.9	15551.0	40543.6
180	No reinfected	<40	Female	53698.3	32990.5	87744.7
380	Reinfected	<40	Female	100232.2	69087.4	145281.7
380	No reinfected	<40	Female	131254.4	89912.8	192731.6
80	Reinfected	>40-60	Female	81449.2	44527.0	147529.8
51	No reinfected	>40-60	Female	168985.8	88671.5	318856.5
180	Reinfected	>40-60	Female	23272.5	14909.2	36358.3
180	No reinfected	>40-60	Female	49956.0	32139.6	78078.9
380	Reinfected	>40-60	Female	93247.0	67275.2	129474.3
380	No reinfected	>40-60	Female	122107.2	87850.8	169110.2
80	Reinfected	>60	Female	171591.1	79196.9	368147.1
51	No reinfected	>60	Female	356007.0	166804.2	767560.8
180	Reinfected	>60	Female	49028.9	25784.8	93815.8
180	No reinfected	>60	Female	105243.7	55407.5	202605.7
380	Reinfected	>60	Female	196446.0	110858.9	350097.5
380	No reinfected	>60	Female	257246.5	146377.5	451441.5
80	Reinfected	<40	Male	48864.3	22343.5	109088.4
51	No reinfected	<40	Male	101380.8	45127.4	226009.9
180	Reinfected	<40	Male	13962.1	7006.0	27840.6
180	No reinfected	<40	Male	29970.5	15425.0	58638.6
380	Reinfected	<40	Male	55942.3	30273.7	103933.0
380	No reinfected	<40	Male	73256.6	40117.1	133362.6
80	Reinfected	>40-60	Male	45459.0	20969.4	98205.7
51	No reinfected	>40-60	Male	94315.5	43416.6	202716.3
180	Reinfected	>40-60	Male	12989.0	6671.8	25438.9
180	No reinfected	>40-60	Male	27881.8	14687.0	52540.7

380	Reinfected	>40-60	Male	52043.7	28700.9	94091.8
380	No reinfected	>40-60	Male	68151.3	38671.9	119777.9
80	Reinfected	>60	Male	95769.6	39188.8	236337.0
51	No reinfected	>60	Male	198697.0	85250.8	469876.2
180	Reinfected	>60	Male	27364.3	12652.1	60081.8
180	No reinfected	>60	Male	58739.3	27466.4	124462.5
380	Reinfected	>60	Male	109641.8	53297.7	224660.3
380	No reinfected	>60	Male	143576.2	71704.9	288173.2

[a] The day from 1st vaccine is selected based on the neutralizing antibody peak after second dose of vaccination, waning period and at the median days from first vaccine dose at the 12-month round (as the neutralizing antibody levels plateaued) according to the reinfection status, age group, and sex

CI: Confidence of interval

Source data are provided as a Source Data file

Supplementary Table 8. Predicted mean probability of positive IgA responses, represented in percentage, in individuals after the administration first vaccination dose according to reinfection status, age group, and sex

Days from 1st vaccine [a]	Reinfection status	Age Group	Sex	Geometric mean	CI 2.5%	CI 97.5%
51	Reinfected	<40	Female	94.7	78.7	99.6
45	No reinfected	<40	Female	99.6	97.2	100.0
180	Reinfected	<40	Female	32.4	5.9	74.0
180	No reinfected	<40	Female	76.3	34.1	97.0
380	Reinfected	<40	Female	84.0	51.4	98.0
380	No reinfected	<40	Female	86.5	54.3	98.6
55	Reinfected	>40-60	Female	97.5	89.6	99.8
47	No reinfected	>40-60	Female	99.8	98.8	100.0
180	Reinfected	>40-60	Female	40.2	11.8	75.4
180	No reinfected	>40-60	Female	83.0	50.6	97.4
380	Reinfected	>40-60	Female	79.7	49.3	95.7
380	No reinfected	>40-60	Female	83.2	54.9	96.9
52	Reinfected	>60	Female	87.1	37.6	99.7
46	No reinfected	>60	Female	98.9	92.2	100.0
180	Reinfected	>60	Female	47.0	3.7	95.2
180	No reinfected	>60	Female	81.4	24.9	99.5
380	Reinfected	>60	Female	94.1	57.9	100.0
380	No reinfected	>60	Female	95.2	65.6	100.0
51	Reinfected	<40	Male	89.3	52.6	99.6
46	No reinfected	<40	Male	99.0	93.0	100.0
180	Reinfected	<40	Male	25.0	1.6	77.4
180	No reinfected	<40	Male	64.6	13.6	97.2
380	Reinfected	<40	Male	73.5	22.2	98.1
380	No reinfected	<40	Male	77.2	25.4	98.7
55	Reinfected	>40-60	Male	94.2	71.3	99.8
47	No reinfected	>40-60	Male	99.5	96.8	100.0
180	Reinfected	>40-60	Male	30.9	3.0	80.2
180	No reinfected	>40-60	Male	72.1	22.2	97.6

380	Reinfected	>40-60	Male	67.5	19.2	96.6
380	No reinfected	>40-60	Male	71.9	23.0	97.4
53	Reinfected	>60	Male	79.8	18.3	99.7
46	No reinfected	>60	Male	97.7	82.0	100.0
180	Reinfected	>60	Male	37.8	1.5	94.3
180	No reinfected	>60	Male	72.9	11.7	99.4
380	Reinfected	>60	Male	90.3	37.8	99.9
380	No reinfected	>60	Male	92.0	46.2	100.0

[a] The day from 1st vaccine is selected based on the IgA peak after second dose of vaccination, waning period and at the median days from first vaccine dose at the 12-month round (as the probability of positive IgA responses plateaued) according to the reinfection status, age group, and sex

CI: Confidence of interval

Source data are provided as a Source Data file

Supplementary Table 9. Predicted mean IFN-γ levels, represented in mIU/ml, in individuals after the administration first vaccination dose according to reinfection status, age group, and sex

Days from 1st vaccine [a]	Reinfection status	Age Group	Sex	Geometric mean	CI 2.5%	CI 97.5%
210	Reinfected	<40	Female	1429.6	673.9	2995.9
210	No reinfected	<40	Female	4465.1	2252.0	9064.3
380	Reinfected	<40	Female	2561.2	1460.8	4448.1
380	No reinfected	<40	Female	4455.3	2601.0	7811.4
210	Reinfected	>40-60	Female	1329.3	631.8	2766.2
210	No reinfected	>40-60	Female	4151.8	2092.5	8263.8
380	Reinfected	>40-60	Female	2381.5	1370.9	4117.1
380	No reinfected	>40-60	Female	4142.7	2403.9	7146.2
210	Reinfected	>60	Female	2604.5	923.4	7534.1
210	No reinfected	>60	Female	8134.9	3136.2	21655.2
380	Reinfected	>60	Female	4666.2	1799.7	11904.4
380	No reinfected	>60	Female	8117.0	3295.7	19942.3
210	Reinfected	<40	Male	1574.4	463.7	5304.6
210	No reinfected	<40	Male	4917.3	1660.7	14774.9
380	Reinfected	<40	Male	2820.6	943.1	8329.5
380	No reinfected	<40	Male	4906.5	1747.2	14043.4
210	No reinfected	>40-60	Male	4572.3	1414.1	14689.7
380	No reinfected	>40-60	Male	4562.2	1511.1	13859.6
210	No reinfected	>60	Male	8958.7	2420.5	32962.0
380	No reinfected	>60	Male	8939.0	2504.4	31698.9

[a] The day from 1st vaccine is selected based on the median of days from the first vaccine at 6- and 12-month rounds according to the reinfection status, age group, and sex
CI: Confidence of interval
Source data are provided as a Source Data file

Supplementary Table 10. Predicted mean IgG levels, represented in AU/ml, in individuals after the administration first vaccination dose according to future infection status, age group, and sex

Days from 1st vaccine [a]	Future infectious status	Age Group	Sex	Geometric mean	CI 2.5%	CI 97.5%
77	Future omicron infected	<40	Female	24845.6	20393.7	30119.5
79	Infection-naïve	<40	Female	27074.8	21903.0	33387.8
180	Future omicron infected	<40	Female	2313.5	1951.7	2752.1
180	Infection-naïve	<40	Female	2455.1	2055.7	2940.3
413	Future omicron infected	<40	Female	15426.4	11169.0	21507.8
389	Infection-naïve	<40	Female	16404.5	14077.5	19134.0
82	Future omicron infected	>40-60	Female	23369.3	20313.9	26980.0
83	Infection-naïve	>40-60	Female	25712.4	22368.2	29591.7
180	Future omicron infected	>40-60	Female	2032.9	1817.3	2279.0
180	Infection-naïve	>40-60	Female	2157.4	1916.4	2418.3
387	Future omicron infected	>40-60	Female	13081.2	11805.0	14463.9
378	Infection-naïve	>40-60	Female	14948.8	13602.2	16429.2
85	Future omicron infected	>60	Female	20726.7	17021.2	25161.3
86	Infection-naïve	>60	Female	22921.3	18955.0	27633.6
180	Future omicron infected	>60	Female	1542.6	1314.4	1807.9
180	Infection-naïve	>60	Female	1637.1	1415.5	1895.1
382	Future omicron infected	>60	Female	13509.9	11851.1	15409.6
375	Infection-naïve	>60	Female	15615.9	13809.1	17642.4
75	Future omicron infected	<40	Male	16896.3	12692.4	22432.2
78	Infection-naïve	<40	Male	18342.3	13539.6	24556.6
180	Future omicron infected	<40	Male	1984.6	1546.3	2576.2
180	Infection-naïve	<40	Male	2106.2	1631.2	2733.3
399*	Future omicron infected	<40	Male	17769.2	12900.2	24430.3
399	Infection-naïve	<40	Male	18906.9	13928.4	25678.5
81	Future omicron infected	>40-60	Male	15773.0	12118.6	20535.6
83	Infection-naïve	>40-60	Male	17330.7	13318.4	22340.0
180	Future omicron infected	>40-60	Male	1743.9	1388.2	2203.3
180	Infection-naïve	>40-60	Male	1850.7	1477.8	2325.4

395	Future omicron infected	>40-60	Male	14956.9	11691.4	19085.3
382	Infection-naïve	>40-60	Male	16701.0	14045.4	19846.7
85	Future omicron infected	>60	Male	13951.6	10556.7	18498.6
86	Infection-naïve	>60	Male	15421.6	11786.5	20116.0
180	Future omicron infected	>60	Male	1323.4	1042.9	1684.0
180	Infection-naïve	>60	Male	1404.4	1112.5	1756.7
387	Future omicron infected	>60	Male	15243.5	12424.4	18650.1
379	Infection-naïve	>60	Male	17350.1	14571.5	20749.6

[a] The day from 1st vaccine is selected based on the IgG peak after the second dose of vaccination, waning period and the IgG peak after the third dose of vaccination according to the reinfection status, age group, and sex

* IgG peak after the third dose did not plateau. Instead, the selected day was chosen based on the IgG peak after the third dose in the infection-naïve group to allow a direct comparison between groups

CI: Confidence of interval

Source data are provided as a Source Data file

Supplementary Table 11. Predicted mean neutralizing antibody levels, represented in IU/ml, in individuals after the administration first vaccination dose according to future infection status, age group, and sex

Days from 1st vaccine [a]	Future infectious status	Age Group	Sex	Geometric mean	CI 2.5%	CI 97.5%
83	Future omicron infected	<40	Female	14410.4	10802.3	19255.0
85	Infection-naïve	<40	Female	15710.4	11433.8	21564.4
180	Future omicron infected	<40	Female	5380.2	4340.5	6686.6
180	Infection-naïve	<40	Female	4602.8	3656.1	5792.9
364	Future omicron infected	<40	Female	62278.5	48659.4	80607.5
367	Infection-naïve	<40	Female	68806.1	53509.2	88798.3
58	Future omicron infected	>40-60	Female	8786.2	7597.7	10175.2
70	Infection-naïve	>40-60	Female	8914.4	7611.9	10414.4
180	Future omicron infected	>40-60	Female	4705.6	4061.0	5439.9
180	Infection-naïve	>40-60	Female	4025.7	3465.4	4671.5
370	Future omicron infected	>40-60	Female	55018.1	47094.9	64153.0
373	Infection-naïve	>40-60	Female	61277.8	53185.2	70899.1
87	Future omicron infected	>60	Female	8266.8	6266.9	10948.6
88	Infection-naïve	>60	Female	9041.1	6769.3	12121.5
180	Future omicron infected	>60	Female	3067.7	2508.3	3748.0
180	Infection-naïve	>60	Female	2624.4	2163.3	3162.9
366	Future omicron infected	>60	Female	54222.4	43165.0	67953.8
369	Infection-naïve	>60	Female	60114.3	49266.9	73359.1
85	Future omicron infected	<40	Male	10918.9	7041.8	16840.1
87	Infection-naïve	<40	Male	11926.1	7731.6	18212.8
180	Future omicron infected	<40	Male	3570.2	2570.6	4990.9
180	Infection-naïve	<40	Male	3054.3	2181.3	4275.1
373	Future omicron infected	<40	Male	62268.7	46830.0	83486.2
375	Infection-naïve	<40	Male	69519.7	52750.1	91999.4
67	Future omicron infected	>40-60	Male	6233.9	4758.1	8243.8
75	Infection-naïve	>40-60	Male	6586.1	4908.6	8856.1
180	Future omicron infected	>40-60	Male	3122.5	2328.2	4194.0
180	Infection-naïve	>40-60	Male	2671.3	1998.1	3570.9

383	Future omicron infected	>40-60	Male	57023.2	44302.5	72917.5
384	Infection-naïve	>40-60	Male	64111.1	50441.2	80947.8
88	Future omicron infected	>60	Male	6287.4	4214.6	9333.0
89	Infection-naïve	>60	Male	6881.5	4776.9	9940.0
180	Future omicron infected	>60	Male	2035.6	1494.0	2763.1
180	Infection-naïve	>60	Male	1741.5	1288.9	2329.3
375	Future omicron infected	>60	Male	54801.2	41925.0	71436.2
377	Infection-naïve	>60	Male	61281.6	47630.3	78411.3

[a] The day from 1st vaccine is selected based on the neutralizing antibody peak after the second dose of vaccination, waning period and the neutralizing antibody peak after the third dose of vaccination according to the reinfection status, age group, and sex

CI: Confidence of interval

Source data are provided as a Source Data file

Supplementary Table 12. Predicted mean probability of positive IgA responses, represented in percentage, in individuals after the administration first vaccination dose according to future infection status, age group, and sex

Days from 1st vaccine [a]	Future infectious status	Age Group	Sex	Geometric mean	CI 2.5%	CI 97.5%
76	Future omicron infected	<40	Female	64.7	54.3	74.2
71	Infection-naïve	<40	Female	56.3	45.9	66.6
180	Future omicron infected	<40	Female	5.2	3.1	8.2
180	Infection-naïve	<40	Female	4.6	2.6	7.3
364	Future omicron infected	<40	Female	32.2	22.8	43.1
359	Infection-naïve	<40	Female	45.9	33.9	58.4
76	Future omicron infected	>40-60	Female	65.5	57.0	73.5
71	Infection-naïve	>40-60	Female	57.2	48.8	65.3
180	Future omicron infected	>40-60	Female	5.3	3.4	7.9
180	Infection-naïve	>40-60	Female	4.7	2.9	6.9
364	Future omicron infected	>40-60	Female	32.9	24.6	42.2
359	Infection-naïve	>40-60	Female	46.7	36.4	57.3
76	Future omicron infected	>60	Female	61.4	51.2	71.2
71	Infection-naïve	>60	Female	52.8	43.2	62.5
180	Future omicron infected	>60	Female	4.5	2.7	7.1
180	Infection-naïve	>60	Female	4.0	2.4	6.1
364	Future omicron infected	>60	Female	29.2	20.5	39.1
359	Infection-naïve	>60	Female	42.4	31.7	53.7
76	Future omicron infected	<40	Male	62.2	48.6	74.4
71	Infection-naïve	<40	Male	53.7	40.1	66.8
180	Future omicron infected	<40	Male	4.8	2.5	8.1
180	Infection-naïve	<40	Male	4.2	2.1	7.2
364	Future omicron infected	<40	Male	30.1	19.0	43.5
359	Infection-naïve	<40	Male	43.3	29.2	58.2
76	Future omicron infected	>40-60	Male	63.0	50.8	74.3
71	Infection-naïve	>40-60	Male	54.5	42.1	66.3
180	Future omicron infected	>40-60	Male	4.9	2.7	8.2
180	Infection-naïve	>40-60	Male	4.3	2.4	7.0

364	Future omicron infected	>40-60	Male	30.7	20.1	43.2
359	Infection-naïve	>40-60	Male	44.1	30.8	58.2
76	Future omicron infected	>60	Male	58.8	45.7	71.1
71	Infection-naïve	>60	Male	50.2	37.7	62.6
180	Future omicron infected	>60	Male	4.2	2.2	7.1
180	Infection-naïve	>60	Male	3.6	1.9	6.0
364	Future omicron infected	>60	Male	27.2	17.2	39.4
359	Infection-naïve	>60	Male	39.9	27.2	53.8

[a] The day from 1st vaccine is selected based on the IgA peak after the second dose of vaccination, waning period and the IgA peak after the third dose of vaccination according to the reinfection status, age group, and sex

CI: Confidence of interval

Source data are provided as a Source Data file

Supplementary Table 13. Predicted mean IFN-γ levels, represented in mIU/ml, in individuals after the administration first vaccination dose according to future infection status, age group, and sex

Days from 1st vaccine [a]	Future infectious status	Age Group	Sex	Geometric mean	CI 2.5%	CI 97.5%
210	Future omicron infected	<40	Female	490.0	358.8	666.5
210	Infection-naïve	<40	Female	743.2	554.8	1001.0
380	Future omicron infected	<40	Female	1586.6	1277.8	1959.1
380	Infection-naïve	<40	Female	1519.0	1217.7	1897.5
210	Future omicron infected	>40-60	Female	356.6	271.4	469.7
210	Infection-naïve	>40-60	Female	540.8	422.1	697.0
380	Future omicron infected	>40-60	Female	1154.6	981.2	1355.9
380	Infection-naïve	>40-60	Female	1105.4	942.1	1295.0
210	Future omicron infected	>60	Female	329.1	240.3	451.3
210	Infection-naïve	>60	Female	499.1	375.5	664.8
380	Future omicron infected	>60	Female	1065.5	858.6	1324.9
380	Infection-naïve	>60	Female	1020.1	826.2	1254.5
210	Future omicron infected	<40	Male	413.6	274.4	612.3
210	Infection-naïve	<40	Male	627.3	430.0	912.7
380	Future omicron infected	<40	Male	1339.3	966.5	1847.3
380	Infection-naïve	<40	Male	1282.2	931.2	1757.3
210	Future omicron infected	>40-60	Male	301.0	206.9	440.4
210	Infection-naïve	>40-60	Male	456.5	323.8	642.9
380	Future omicron infected	>40-60	Male	974.6	725.3	1311.8
380	Infection-naïve	>40-60	Male	933.1	702.8	1225.8
210	Future omicron infected	>60	Male	277.8	186.3	413.3
210	Infection-naïve	>60	Male	421.3	294.0	599.7
380	Future omicron infected	>60	Male	899.4	651.0	1224.2
380	Infection-naïve	>60	Male	861.0	639.2	1161.4

[a] The day from 1st vaccine is selected based on the median of days from the first vaccine at 6- and 12-month rounds according to the future infection status, age group, and sex
CI: Confidence of interval
Source data are provided as a Source Data file

Supplementary Table 14. Predicted mean IgG levels, represented in AU/ml, in individuals after the administration first vaccination dose according to immune imprinting status, age group, and sex

Days from 1st vaccine [a]	Immune imprinting status	Age Group	Sex	Geometric mean	CI 2.5%	CI 97.5%
48	Before omicron/Reinfected	<40	Female	45324.8	29493.7	69612.9
79	Infected with omicron	<40	Female	18988.7	13805.5	26104.8
180	Before omicron/Reinfected	<40	Female	7446.1	4552.5	12172.1
180	Infected with omicron	<40	Female	2119.7	1614.2	2778.7
387	Before omicron/Reinfected	<40	Female	19486.1	14070.5	27085.9
407	Infected with omicron	<40	Female	43958.0	34770.3	55826.7
50	Before omicron/Reinfected	>40-60	Female	34200.7	22056.5	53057.4
82	Infected with omicron	>40-60	Female	16133.9	12331.2	21142.3
180	Before omicron/Reinfected	>40-60	Female	6343.8	3928.3	10285.3
180	Infected with omicron	>40-60	Female	1806.0	1449.3	2246.4
380*	Before omicron/Reinfected	>40-60	Female	19131.6	13235.0	27610.9
380*	Infected with omicron	>40-60	Female	38634.7	32693.9	45610.6
54	Before omicron/Reinfected	>60	Female	32388.7	18224.0	58045.4
84	Infected with omicron	>60	Female	18332.4	11121.5	30132.3
180	Before omicron/Reinfected	>60	Female	6723.2	3551.4	12888.9
180	Infected with omicron	>60	Female	1914.0	1186.4	3116.6
370	Before omicron/Reinfected	>60	Female	22356.7	12961.0	38165.1
391	Infected with omicron	>60	Female	45804.0	33216.4	63704.9
54	Before omicron/Reinfected	<40	Male	33186.6	19098.9	57725.4
84	Infected with omicron	<40	Male	18859.3	11474.1	31168.4
180	Before omicron/Reinfected	<40	Male	6175.4	3361.4	11521.4
180	Infected with omicron	<40	Male	1758.0	1142.7	2694.4
388	Before omicron/Reinfected	<40	Male	16762.0	11344.6	24656.8
406	Infected with omicron	<40	Male	37747.7	25801.2	54900.1
57	Before omicron/Reinfected	>40-60	Male	25880.1	14648.6	45472.8
86	Infected with omicron	>40-60	Male	16175.4	9853.7	26517.6
180	Before omicron/Reinfected	>40-60	Male	5261.3	2956.7	9611.8
180	Infected with omicron	>40-60	Male	1497.8	1036.5	2172.7

380*	Before omicron/Reinfected	>40-60	Male	16424.6	10752.0	25210.8
380*	Infected with omicron	>40-60	Male	33168.3	25168.4	43779.3
67	Before omicron/Reinfected	>60	Male	26519.4	12448.4	57851.3
87	Infected with omicron	>60	Male	18539.3	9778.0	35430.8
180	Before omicron/Reinfected	>60	Male	5575.9	2740.7	11333.2
180	Infected with omicron	>60	Male	1587.4	920.7	2748.9
372	Before omicron/Reinfected	>60	Male	19109.1	11053.1	32924.1
391	Infected with omicron	>60	Male	39413.6	27149.5	57548.4

[a] The day from 1st vaccine is selected based on the IgG levels at the waning period and the IgG peak after the third dose of vaccination according to the immune imprinting status, age group, and sex

* IgG peak after the third dose did not plateau. Instead, the median days from the 12-months sampling round was selected.

CI: Confidence of interval

Source data are provided as a Source Data file

Supplementary Table 15. Predicted mean neutralizing antibody levels, represented in IU/ml, in individuals after the administration first vaccination dose according to immune imprinting status, age group, and sex

Days from 1st vaccine [a]	Immune imprinting status	Age Group	Sex	Geometric mean	CI 2.5%	CI 97.5%
43	Before omicron/Reinfected	<40	Female	146500.6	47648.5	452401.5
83	Infected with omicron	<40	Female	8980.3	6587.0	12319.2
180	Before omicron/Reinfected	<40	Female	36049.4	19569.4	66900.2
180	Infected with omicron	<40	Female	4210.8	3153.6	5590.6
403*	Before omicron/Reinfected	<40	Female	161599.8	90048.9	284183.1
403	Infected with omicron	<40	Female	278283.8	204666.8	371499.7
43	Before omicron/Reinfected	>40-60	Female	117992.6	38568.2	365904.0
83	Infected with omicron	>40-60	Female	7232.8	5411.6	9592.6
180	Before omicron/Reinfected	>40-60	Female	29034.4	15705.8	53990.1
180	Infected with omicron	>40-60	Female	3391.4	2618.2	4380.3
403*	Before omicron/Reinfected	>40-60	Female	130153.6	72007.4	229584.5
403	Infected with omicron	>40-60	Female	224131.8	169729.8	291023.5
43	Before omicron/Reinfected	>60	Female	135619.4	43189.7	426974.0
83	Infected with omicron	>60	Female	8313.3	5445.4	12675.0
180	Before omicron/Reinfected	>60	Female	33371.8	16936.2	65804.7
180	Infected with omicron	>60	Female	3898.0	2596.4	5869.2
403*	Before omicron/Reinfected	>60	Female	149597.1	77403.9	284571.3
403	Infected with omicron	>60	Female	257614.5	168280.0	392277.1
43	Before omicron/Reinfected	<40	Male	128061.0	41250.3	404999.9
83	Infected with omicron	<40	Male	7850.0	5308.2	11658.5
180	Before omicron/Reinfected	<40	Male	31512.0	16282.9	61206.7
180	Infected with omicron	<40	Male	3680.8	2520.0	5365.1
403*	Before omicron/Reinfected	<40	Male	141259.7	75906.2	261823.8
403	Infected with omicron	<40	Male	243257.1	163183.8	353462.1
43	Before omicron/Reinfected	>40-60	Male	103141.2	33055.9	321986.2
83	Infected with omicron	>40-60	Male	6322.4	4334.0	9181.2
180	Before omicron/Reinfected	>40-60	Male	25380.0	13009.4	49711.3
180	Infected with omicron	>40-60	Male	2964.5	2071.0	4230.0

403*	Before omicron/Reinfected	>40-60	Male	113771.6	60643.6	212204.3
403	Infected with omicron	>40-60	Male	195921.0	135413.2	281352.3
43	Before omicron/Reinfected	>60	Male	118549.4	37259.9	381681.1
83	Infected with omicron	>60	Male	7267.0	4552.6	11676.3
180	Before omicron/Reinfected	>60	Male	29171.4	14075.5	60326.6
180	Infected with omicron	>60	Male	3407.4	2151.4	5425.5
403*	Before omicron/Reinfected	>60	Male	130767.7	66128.0	260162.2
403	Infected with omicron	>60	Male	225189.3	138798.4	361099.7

[a] The day from 1st vaccine is selected based on the neutralizing antibody levels at the waning period and the neutralizing antibody peak after the third dose of vaccination according to the immune imprinting status, age group, and sex

* Neutralizing antibody peak after the third dose did not plateau. Instead, the selected day was chosen based on the neutralizing antibody peak after the third dose in the "infected with omicron" group to allow a direct comparison between groups

CI: Confidence of interval

Source data are provided as a Source Data file

Supplementary Table 16. Predicted mean probability of positive IgA responses, represented in percentage, in individuals after the administration first vaccination dose according to immune imprinting status, age group, and sex

Days from 1st vaccine [a]	Immune imprinting status	Age Group	Sex	Geometric mean	CI 2.5%	CI 97.5%
58	Before omicron/Reinfected	<40	Female	95.6	80.0	99.7
71	Infected with omicron	<40	Female	39.5	20.5	61.6
180	Before omicron/Reinfected	<40	Female	43.1	10.8	81.6
180	Infected with omicron	<40	Female	1.5	0.3	4.8
410	Before omicron/Reinfected	<40	Female	90.7	62.4	99.3
415	Infected with omicron	<40	Female	98.4	93.5	99.8
59	Before omicron/Reinfected	>40-60	Female	94.9	75.9	99.7
71	Infected with omicron	>40-60	Female	35.5	19.5	54.5
180	Before omicron/Reinfected	>40-60	Female	39.5	8.8	79.3
180	Infected with omicron	>40-60	Female	1.3	0.2	4.0
411	Before omicron/Reinfected	>40-60	Female	89.3	58.0	99.3
415	Infected with omicron	>40-60	Female	98.2	93.0	99.8
62	Before omicron/Reinfected	>60	Female	80.8	32.7	98.8
71	Infected with omicron	>60	Female	11.2	2.3	31.1
180	Before omicron/Reinfected	>60	Female	14.4	1.3	50.3
180	Infected with omicron	>60	Female	0.3	0.0	1.4
436	Before omicron/Reinfected	>60	Female	69.1	6.0	99.6
421	Infected with omicron	>60	Female	91.3	63.7	99.4
57	Before omicron/Reinfected	<40	Male	98.1	89.8	99.9
71	Infected with omicron	<40	Male	63.3	34.7	86.5
180	Before omicron/Reinfected	<40	Male	64.1	21.5	94.1
180	Infected with omicron	<40	Male	4.2	0.7	13.1
407	Before omicron/Reinfected	<40	Male	95.7	79.2	99.8
415	Infected with omicron	<40	Male	99.3	96.8	100.0
58	Before omicron/Reinfected	>40-60	Male	97.7	88.2	99.9
71	Infected with omicron	>40-60	Male	59.6	33.1	82.5
180	Before omicron/Reinfected	>40-60	Male	60.8	18.8	92.9
180	Infected with omicron	>40-60	Male	3.5	0.6	10.9

408	Before omicron/Reinfected	>40-60	Male	95.1	75.6	99.7
415	Infected with omicron	>40-60	Male	99.2	96.5	100.0
60	Before omicron/Reinfected	>60	Male	90.3	54.7	99.6
71	Infected with omicron	>60	Male	25.1	5.5	58.9
180	Before omicron/Reinfected	>60	Male	29.1	3.2	76.3
180	Infected with omicron	>60	Male	0.9	0.1	3.6
416	Before omicron/Reinfected	>60	Male	81.9	29.2	99.3
419	Infected with omicron	>60	Male	96.2	81.4	99.8

[a] The day from 1st vaccine is selected based on the probability of positive IgA responses at the waning period and the probability of positive IgA responses peak after the third dose of vaccination according to the immune imprinting status, age group, and sex

CI: Confidence of interval

Source data are provided as a Source Data file

Supplementary Table 17. Predicted mean IFN-γ levels, represented in mIU/ml, in individuals after the administration first vaccination dose according to immune imprinting status, age, and sex

Days from 1st vaccine [a]	Immune imprinting status	Age Group	Sex	Geometric mean	CI 2.5%	CI 97.5%
210	Before omicron/Reinfected	<40	Female	5260.2	1498.3	18145.3
210	Infected with omicron	<40	Female	641.9	413.7	989.9
380	Before omicron/Reinfected	<40	Female	4950.7	2821.8	8806.2
380	Infected with omicron	<40	Female	4172.8	2984.6	5846.8
210	Before omicron/Reinfected	>40-60	Female	4545.6	1314.1	15632.8
210	Infected with omicron	>40-60	Female	554.7	374.5	831.7
380	Before omicron/Reinfected	>40-60	Female	4278.1	2387.0	7862.2
380	Infected with omicron	>40-60	Female	3606.0	2748.5	4750.4
210	Before omicron/Reinfected	>60	Female	3435.1	907.7	12539.5
210	Infected with omicron	>60	Female	419.2	211.8	830.4
380	Before omicron/Reinfected	>60	Female	3232.9	1462.8	7323.2
380	Infected with omicron	>60	Female	2725.0	1478.0	5008.4
210	Before omicron/Reinfected	<40	Male	3919.6	1025.9	14779.1
210	Infected with omicron	<40	Male	478.3	270.4	845.3
380	Before omicron/Reinfected	<40	Male	3688.9	1808.2	7645.5
380	Infected with omicron	<40	Male	3109.3	1896.9	5067.6
210	Infected with omicron	>40-60	Male	413.3	238.6	720.0
380	Infected with omicron	>40-60	Male	2686.9	1687.7	4235.0
210	Infected with omicron	>60	Male	312.4	149.2	668.0
380	Infected with omicron	>60	Male	2030.5	1014.9	4022.6

[a] The day from 1st vaccine is selected based on the median of days from the first vaccine at 6- and 12-month rounds according to the future infection status, age group, and sex
CI: Confidence of interval
Source data are provided as a Source Data file

Supplementary Table 18. Multiple linear regression analysis for IgG, IgA, neutralizing antibody (nAb), and IFN-γ levels associations with sex, age, time from last vaccine dose administrated or infection, and the infection status in the different cohorts studied (reinfected, future infection, and immune imprinting cohorts). Data are expressed in log10(AU/ml) for IgG and IgA, log10(IU/ml) for nAb, and log10(mIU/ml) for IFN-γ.

Reinfected cohort												
	IgG log10(AU/ml)			IgA log10(AU/ml)			Neutralizing antibodies log10(IU/ml)			IFN-γ log10(mIU/ml) [a]		
Variable	Estimate	Std. Error	p-value	Estimate	Std. Error	p-value	Estimate	Std. Error	p-value	Estimate	Std. Error	p-value
Intercept [b]	4.8925	0.0721	<2e-16***	3.2943	0.2227	<2e-16***	5.5116	0.1515	<2e-16***	N.A.	N.A.	N.A.
Age, >40–60 years old	0.0021	0.0646	0.9731	0.0081	0.1995	0.9677	-0.0770	0.1348	0.5688	N.A.	N.A.	N.A.
Age, >60 years old	0.2428	0.1018	0.0188*	-0.0464	0.3143	0.8829	0.0600	0.2114	0.7772	N.A.	N.A.	N.A.
Sex, male	-0.1175	0.0929	0.2089	-0.2162	0.2868	0.4525	-0.3792	0.1930	0.0522	N.A.	N.A.	N.A.
Time from second vaccine dose (days)	-0.0068	0.0006	<2e-16***	-0.0140	0.0020	4.06e-10***	-0.0064	0.0014	2.14e-05***	N.A.	N.A.	N.A.
Reinfected status, Reinfected	-0.2072	0.0592	0.0006***	-0.3766	0.1826	0.0416*	-0.2647	0.1240	0.0351*	N.A.	N.A.	N.A.

Future infection cohort												
	IgG log10(AU/ml)			IgA log10(AU/ml)			Neutralizing antibodies log10(IU/ml)			IFN-γ log10(mIU/ml)		
Variable	Estimate	Std. Error	p-value	Estimate	Std. Error	p-value	Estimate	Std. Error	p-value	Estimate	Std. Error	p-value
Intercept [c]	4.7117	0.0655	<2e-16***	2.1513	0.2151	<2e-16***	5.4289	0.0896	<2e-16***	3.4840	0.1473	<2e-16***
Age, >40–60 years old	0.0040	0.0318	0.8982	0.1915	0.1045	0.0672	0.0190	0.0435	0.662	-0.1373	0.0637	0.0316*
Age, >60 years old	0.0261	0.0363	0.4715	0.2623	0.1192	0.0281	0.0134	0.0497	0.787	-0.2491	0.0737	0.0007***
Sex, male	0.0677	0.0362	0.0617	-0.1195	0.1189	0.3150	0.0513	0.0497	0.302	-0.0376	0.0733	0.6077
Time from booster (days)	-0.0066	0.0007	<2e-16***	-0.0128	0.0024	2.17e-07***	-0.0078	0.0010	5.25e-14***	-0.0040	0.0017	0.0220*
Future infected status, Infected	-0.0574	0.0241	0.0178*	-0.1635	0.0793	0.0397*	-0.0541	0.0331	0.103	0.0061	0.0497	0.9023

Immune imprinting cohort												
	IgG log10(AU/ml)			IgA log10(AU/ml)			Neutralizing antibodies log10(IU/ml)			IFN-γ log10(mIU/ml)		
**Variable**	**Estimate**	**Std. Error**	**p-value**	**Estimate**	**Std. Error**	**p-value**	**Estimate**	**Std. Error**	**p-value**	**Estimate**	**Std. Error**	**p-value**
Intercept [d]	4.1268	0.0777	<2e-16***	1.8431	0.2366	5.32e-13***	4.9596	0.1241	<2e-16***	3.6388	0.1638	<2e-16***
Age, >40–60 years old	0.0156	0.0480	0.7445	-0.0038	0.1462	0.9788	0.0084	0.0767	0.9125	-0.0892	0.0926	0.337
Age, >60 years old	0.0419	0.0763	0.5834	-0.2714	0.2325	0.2445	0.0567	0.1243	0.6486	-0.1697	0.1543	0.274
Sex, male	-0.1125	0.0565	0.0482*	0.0811	0.1722	0.6379	-0.0782	0.0904	0.3881	-0.1128	0.1104	0.309
Time from last infection (days)	0.0078	0.0018	2.19-0.5***	0.0203	0.0054	0.0002***	0.0086	0.0028	0.0031*	0.0037	0.0055	0.507
Infected status, Infected with Omicron	0.3069	0.0683	1.25e-05***	0.4394	0.2079	0.0359*	0.2106	0.1090	0.0549*	-0.0565	0.1362	0.679

[a] Intercept defined as a <40 years old female individual, not reinfected, with IgG/IgA/nAb/IFN-γ levels at 14 days after the administration of the second dose
[b] Intercept defined as a <40 years old female individual, not future infected, with IgG/IgA/nAb/IFN-γ levels at 14 days after the administration of the third dose
[a] Intercept defined as a <40 years old female individual, infected before Omicron and reinfected, with IgG/IgA/nAb/IFN-γ levels at 0 days after last infection
* p<0.05, ** p<0.01, **** p<0.0001 (two-sided p-values were calculated using multiple linear regression analysis). p<0.05 was considered significant.
N.A.: Not applicable, small size group
Source data are provided as a Source Data file

Supplementary References

1. Hansen, C. B. *et al.* SARS-CoV-2 Antibody Responses Are Correlated to Disease Severity in COVID-19 Convalescent Individuals. *J. Immunol.* **206**, 109–117 (2021).

2. Bayarri-Olmos, R. *et al.* SARS-CoV-2 Neutralizing Antibody Responses towards Full-Length Spike Protein and the Receptor-Binding Domain. *J. Immunol.* **207**, 878–887 (2021).

3. Brooks, M. *et al.* glmmTMB Balances Speed and Flexibility Among Packages for Zero-inflated Generalized Linear Mixed Modeling. *R J.* **9**, 378–400 (2017).

4. Pérez-Alós, L. *et al.* Modeling of waning immunity after SARS-CoV-2 vaccination and influencing factors. *Nat. Commun.* **13**, 1614 (2022).
